# AND logic nanoparticle for precision immunotherapy of metastatic cancers

**DOI:** 10.1038/s41565-026-02130-3

**Published:** 2026-03-09

**Authors:** Shuyue Ye, Shuang Chen, Vijay Basava, Katy Torres, Yangyang Zhao, Gang Huang, Mingyi Chen, Jinming Gao

**Affiliations:** 1 Department of Biomedical Engineering, University of Texas Southwestern Medical Center, Dallas, TX USA; 2 Simmons Comprehensive Cancer Center, University of Texas Southwestern Medical Center, Dallas, TX USA; 3 Department of Pharmacology, University of Texas Southwestern Medical Center, Dallas, TX USA; 4 Department of Pathology, University of Texas Southwestern Medical Center, Dallas, TX USA; 5 Department of Cell Biology, University of Texas Southwestern Medical Center, Dallas, TX USA

**Keywords:** Nanoparticles, Drug delivery, Bioconjugate chemistry, Polymer synthesis

## Abstract

Success in systemic immunotherapy against metastatic cancer hinges on the ability to achieve tumour-specific immune activation over normal tissues. Single-gate stimuli-responsive systems are not adequate at differentiating tumour versus normal tissue signals. Here we report an AND-gated nanoparticle that requires acidic pH and hypoxia signals to activate the stimulator of interferon genes (STING) pathway in systemic therapy of metastatic cancers. The dual stimuli-responsive nanoparticle consists of a small-molecule STING agonist conjugated to a pH-sensitive polymer through a hypoxia-sensitive linker. Biochemical analyses confirmed the (pH–hypoxia) AND logic truth table in STING activation. The nanoparticle agonist significantly reduced metastatic burdens in multiple immune-cold tumour models while exhibiting minimal systemic toxicity. Mechanistic investigation revealed that STING activation in tumour-resident type I dendritic cells drives CD8^+^ T cell priming and infiltration, which synergizes with immune checkpoint inhibitors. This AND logic nanoplatform offers a safe and efficacious therapeutic for STING-mediated immunotherapy against metastatic cancers.

## Abstract (web summary)

An AND logic nanoparticle designed to respond to both acidic pH and hypoxia signals for immune activation in tumour microenvironment allows safe systemic therapy of metastatic cancers.

## Main

Metastatic disease is the leading cause of cancer mortality, accounting for >90% of cancer-related deaths^1,2^. Conventional treatments (for example, chemotherapy and radiation) provide limited benefit and often cause dose-limiting toxicities^3,4^. In recent years, immunotherapy has changed the landscape of cancer care by harnessing host immune defence against cancer^5^. The stimulator of interferon genes (STING) pathway has emerged as a critical component in the activation of innate and adaptive responses in antitumour immunity^6,7^. Activation of the STING pathway initiates a signalling cascade that organizes immune cells and promotes a multifaceted type I interferon (I-IFN) response that promotes the maturation and migration of dendritic cells (DCs), and primes cytotoxic T lymphocytes and natural killer (NK) cells against tumour cells^8–10^. Despite its therapeutic potential, STING-targeted therapies face significant challenges, including poor tumour specificity and the risk of excessive immune activation in healthy tissues^11,12^.

Extensive research has been dedicated to the design and development of stimuli-responsive strategies to improve the safety and antitumour efficacy of cytotoxic drugs^13,14^. These systems are designed to respond to biological signals that are elevated in tumours over normal tissues^15,16^. While single stimulus-responsive systems have shown promise, they are often inadequate at achieving high tumour specificity because of the heterogeneous and analogue nature of biological signals^17,18^. To overcome these challenges, design of nanoparticles (NPs) that requires multiple stimuli signals to achieve intended pharmacological functions may prove useful. In the electronic industry, advances from single-gate transistors to multi-gate logic devices have transformed data transfer and computation^19^. In chimeric antigen receptor T therapy, logic gate concept has also been introduced in cell circuitry to improve cancer specificity of cytotoxic T cells^20^.

**Fig. 1:**
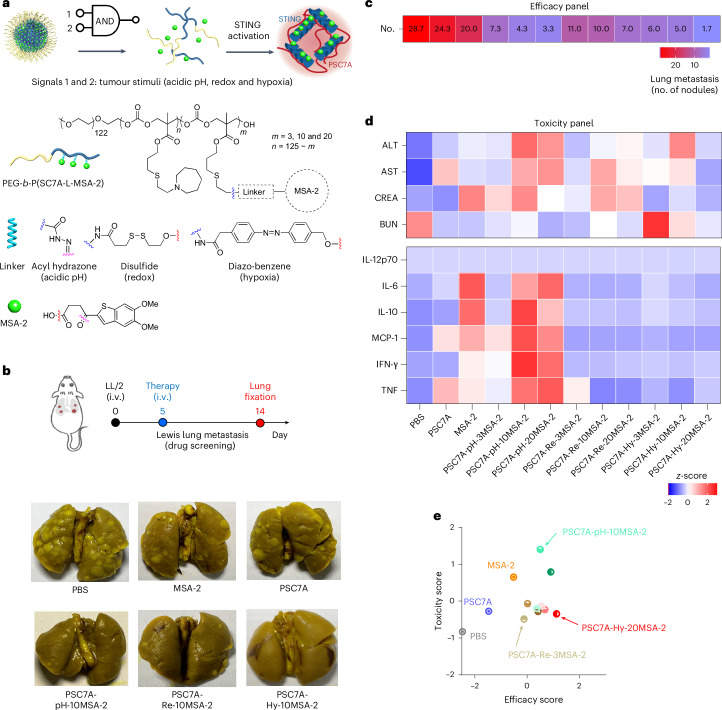
Screening of STING-activating NPs for their safety and antitumour efficacy in an LL/2 lung metastatic model. **a**, Schematic of AND logic NP design and chemical structure of PEG-*b*-P(SC7A-L-MSA-2). **b**, Representative optical images of mouse lungs after single i.v. injection of different STING NPs in mice with LL/2 metastatic nodules. **c**, Number of LL/2 nodules after i.v. injection of PBS, MSA-2 (2.3 mg kg^−1^), PSC7A (20 mg kg^−1^) and AND logic NPs (20 mg kg^−1^) (*n* = 4). **d**, Heat map for the serum liver and kidney toxicity assessment and systemic cytokine levels from mice treated in **c** (*n* = 4). **e**, Evaluation of antitumour efficacy (*x* axis) and toxicity (*y* axis) using dimensionality reduction methods for AND logic NP evaluation. MCP-1, monocyte chemoattractant protein-1; TNF, tumour necrosis factor. Source data

In this study, we introduce the design and evaluation of a dual stimuli-responsive AND logic NP for systemic STING therapy of metastatic cancers. The nanoplatform is built on a proton transistor polymer, poly(ethylene oxide)-*b*-PSC7A (PSC7A, pKa = 6.9, with threshold response to acidic pH (<6.9) and non-canonical STING activation property^21^. A small-molecule STING agonist, 5,6-dimethoxy-γ-oxo-benzo[*b*]thiophene-2-butanoic acid (MSA-2)^22^, is conjugated to the polymer through different linkers that respond to acidic pH, redox or hypoxia signals (Fig. 1a). Using a metastatic Lewis lung carcinoma model (LL/2), we screened different compositions of STING-activating NPs and identified (pH–hypoxia) AND logic NPs with the largest therapeutic window. The resulting NP agonist significantly reduced metastatic burdens in preclinical models of Lewis lung carcinoma, triple-negative breast cancer and melanoma, while exhibiting minimal systemic toxicity.

## Syntheses and screening of stimuli-responsive NPs

We synthesized a series of dual stimuli-responsive polymers by conjugating MSA-2 to PSC7A polymers using different linkers (Fig. 1a). For pH-sensitive linker, we used an acyl hydrazone bond^23^ (Supplementary Fig. 1); for redox-sensitive linker, we introduced a disulfide bond that responds to glutathione (GSH)^24^ (Supplementary Fig. 2); for hypoxia-sensitive linker, we used a diazo-benzene bond that is susceptible to NAD(P)H quinone oxidoreductase 1 (NQO1), an enzyme that is highly elevated under the hypoxic environment of tumours^25,26^ (Supplementary Fig. 3). To ensure ultra-pH sensitivity and sufficient conjugation density, we chose PSC7A polymers with an average repeating unit of 125, and systematically varied drug-to-polymer ratios (DPRs) (3, 10 and 20). After polymer syntheses, we used ^1^H nuclear magnetic resonance (Supplementary Figs. 4–6) and ultraviolet–visible spectroscopy to quantify the number of conjugated MSA-2 to the PSC7A polymers (Extended Data Table 1 and Supplementary Fig. 7). pH titration experiments show that these polymers maintained the ultra-sensitive pH response inherent to the parent PSC7A polymer even when the DPR value reaches 20 for each linker design (Supplementary Fig. 8).

**Extended Data Fig. 1:**
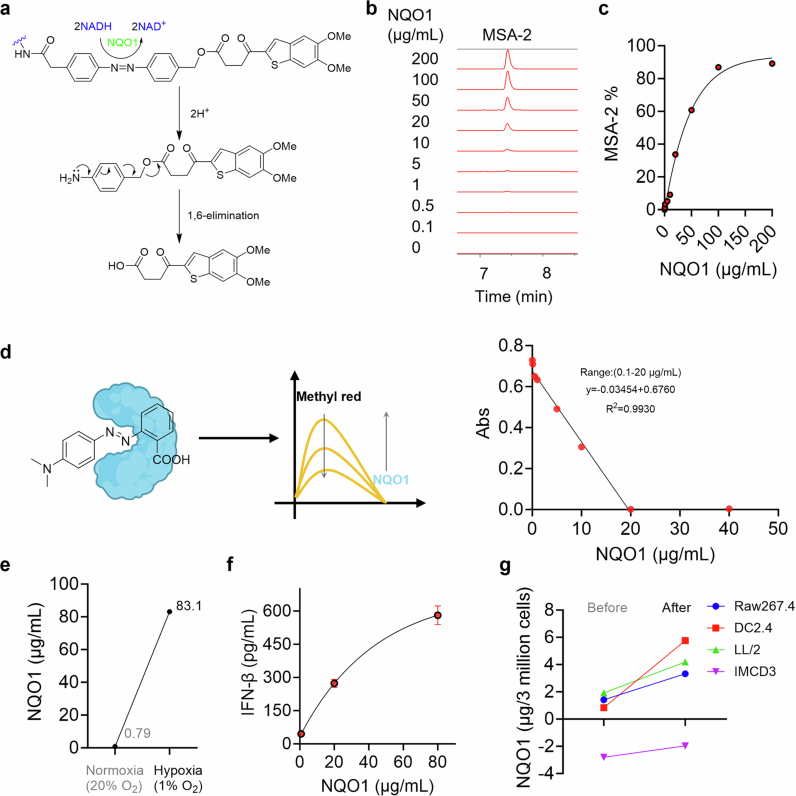
NQO1-mediated release of MSA-2 from PSC7A-Hy-MSA-2 copolymer. **a**, Schematic of MSA-2 release mechanism. Hydride transfer from NADH leads to NQO1-catalyzed reduction of azobenzene bond. The resulting aniline intermediate undergoes a 1,6-elimination reaction, triggering self-immolation to produce cleaved MSA-2. **b-c**, MSA-2 release from PSC7A-Hy-MSA-2 NPs (1 mg/mL, pH = 6.5 PBS) after incubation with varying concentrations of NQO1 and NADH (800 µM) for 20 hours at 37 °C. The HPLC retention time at 7.44 min corresponds to the released MSA-2. **d**, Standard calibration curve of NQO1 activity determined by monitoring the absorbance change of methyl red (25 μg/mL) in the presence of NADH (1 mM) and varying NQO1 concentrations at 430 nm. **e**, Intracellular NQO1 concentrations in DC2.4 cells cultured under 20% or 1% O_2_ for 24 h. Cell lysates were prepared from 3 × 10^5^ cells in 10 μL of lysis buffer. **f**, IFN-β production in DC2.4 cells after 24 h incubation with filtrates generated from reactions containing PHM NP (1 mg/mL), NADH (1 mM), and varying NQO1 concentrations (0.8, 20, or 80 μg/mL). **g**, Quantification of NQO1 activity in RAW264.7, DC2.4, LL/2, and IMCD3 cells before and after 24 h co-incubation with PHM NP (0.1 mg/mL). Source data

**Extended Data Fig. 2:**
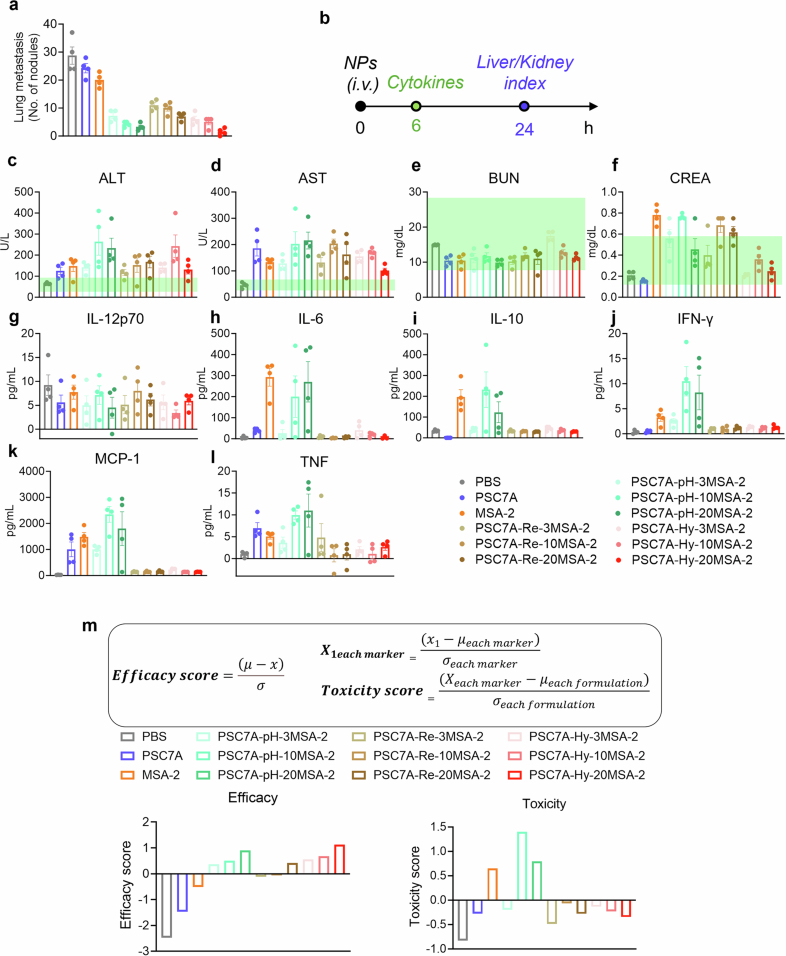
Efficacy and safety evaluation of different STING-activating NPs. **a**, Lung metastasis nodules observed after treatments with PBS, free MSA-2 (2.3 mg/kg), PSC7A polymer (20 mg/kg), and NPs (20 mg/kg) formulated with DPR of 3, 10, and 20. **b**, Schematic of the experimental setup used for efficacy and toxicity assessment (Data represent the mean ± SEM, n = 4 mice per group). **c**-**f**, Levels of ALT, AST, BUN, and CREA following treatment with the indicated formulations and doses. The green shading area denotes the normal range for each toxicity marker (Data represent the mean ± SEM, n = 4 mice per group). **g**-**l**, Cytokine levels, including IL-12p70, IL-6, IL-10, IFN-γ, MCP-1, and TNF, measured after the same treatments (Data represent the mean ± SEM, n = 4 mice per group). **m**, Combined efficacy and toxicity scores calculated using the equations shown: ***μ*** is the average value of the nodule numbers for all formulations; **σ** is the standard deviation of the nodules number for all formulations. ${\boldsymbol{x}}{\boldsymbol{1}}$ are the toxicity values; ${{\boldsymbol{\mu }}}_{{each\; marker}}$ is the mean of the toxicity values for each marker; ${{\boldsymbol{\sigma }}}_{{each\; marker}}$ is the standard deviation of the toxicity values for each marker; ${{\boldsymbol{\mu }}}_{{\boldsymbol{each\; formulation}}}$ is the mean of ${{\rm{X}}}_{{each\; marker}}$ for each formulation; ${{\boldsymbol{\sigma }}}_{{\boldsymbol{each\; formulation}}}$ is the standard deviation of the ${{\rm{X}}}_{{each\; marker}}$ for each formulation. Source data

We used a pH-induced self-assembly method to formulate micelle NPs from the copolymers (Methods). Dynamic light scattering analysis shows comparable hydrodynamic diameter (25–30 nm) with narrow polydispersity (<0.15) across different compositions at pH 7.4 (Extended Data Table 2 and Supplementary Fig. 9). The copolymers showed MSA-2 release upon exposure to the corresponding stimuli (pH, redox or hypoxia) (Extended Data Fig. 1a and Supplementary Fig. 10). To assess the therapeutic efficacy, we utilized an immune-cold LL/2 lung metastasis model. Five days after tumour inoculation, a single injection of NPs (polymer dose 20 mg kg^−1^) or MSA-2 (2.3 mg kg^−1^, equivalent to polymers with DPR of 20) was intravenously injected, and tumour burdens in the lung were evaluated on day 14 (Fig. 1b). Representative optical images of the mouse lungs show the highest number of lung nodules in the phosphate-buffered saline (PBS) control, with a reduced number of nodules in the MSA-2 and PSC7A NP treatments. Quantitative analysis of lung nodules from all the treatment groups (*n* = 4 for each condition) identified (pH–hypoxia) dual-responsive NPs with DPR of 20 yielded the lowest tumour burden (1–2 nodules per lung), in stark contrast to ~30 nodules in the PBS group (Fig. 1c and Extended Data Fig. 2a). To assess the effect of DPR on antitumour efficacy for each polymer design, we performed pairwise two-tailed *t*-tests and found that DPR 20 also led to least tumour burdens in each linker design (Supplementary Fig. 11).

The safety profiles of the NPs were evaluated by measuring liver enzymes, including alanine aminotransferase (ALT) and aspartate aminotransferase (AST), and kidney functions such as creatinine (CREA) and blood urea nitrogen (BUN) 24 h post-injection, as well as systemic cytokine levels (for example, interleukin (IL)-6 and interferon (IFN)-γ) 6 h post-injection (Fig. 1d and Extended Data Figs. 2c–l). Free MSA-2 and NPs with pH-responsive linkers exhibited increased cytokines, while NPs with pH or redox-responsive linkers show high liver enzyme level. NPs with hypoxia-responsive linker and DPR 20 show mild cytokine and liver enzyme levels similar to PBS control.

**Extended Data Fig. 3:**
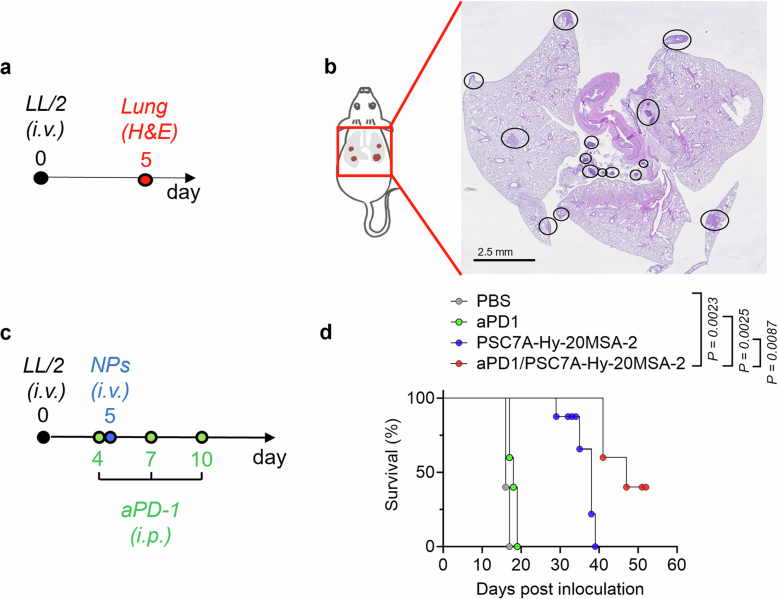
PSC7A-Hy-20MSA-2 NP shows superior efficacy over aPD1 with additional synergy in LL/2 metastatic tumor model. **a**-**b**, Representative H&E images of lung tissues 5 days after intravenous injection of LL/2 cancer cells. Lung nodules are present on Day 5 before NP treatment. **c**, Diagram illustrating the treatment regimen of PSC7A-Hy-20MSA-2 with aPD1. **d**, Survival analysis of mice following treatment with PBS, aPD1, PSC7A-Hy-20MSA-2 (20 mg/kg), and the combination of aPD1 with PSC7A-Hy-20MSA-2 (n = 5 mice per group). P values were determined by the two-sided Mantel–Cox test (**d**). Source data

To identify optimal formulation, antitumour efficacy (nodule count) and safety (toxicity biomarker levels) were calculated into efficacy and toxicity scores, respectively (Extended Data Fig. 2m). Evaluation of both scores revealed PSC7A-Hy-20MSA-2 NP as the lead candidate with the maximal efficacy and reduced toxicity (Fig. 1e). Although PSC7A-Re-3MSA-2 NP had the lowest toxicity, its efficacy score was subpar. Survival studies further illustrated significantly increased mouse longevity by PSC7A-Hy-20MSA-2 NP treatment over anti-programmed cell death protein 1 (aPD1) therapy and synergy in the combination therapy (Extended Data Fig. 3).

We also investigated the performance of PSC7A-Hy-20MSA-2 NP over free MSA-2 and MSA-2-loaded PSC7A NP (PSC7A@MSA-2, physical loading). For MSA-2, we administered either intravenous (i.v.) (2.3 mg kg^−1^ to match the dose from PSC7A-Hy-20MSA-2) or orally (intragastric administration, i.g., 60 mg kg^−1^ based on literature report^22^). Data show that PSC7A-Hy-20MSA-2 NP exhibited the strongest antitumour efficacy with the lowest number of metastatic nodules in the lung (Supplementary Fig. 12a–c). Free MSA-2 (either i.v. or i.g.) did not lead to clear protection compared with PBS control. The PSC7A@MSA-2 group failed to produce high antitumour efficacy and instead triggered cytokine elevation as free MSA-2 controls, suggesting premature release of MSA-2 during circulation. PSC7A-Hy-20MSA-2 NP showed no overt histological injury in the liver or major organs, confirming its superior safety profile (Supplementary Figs. 12d–n and 13). However, free MSA-2 (i.g.) caused mucosal injury characterized by villus blunting, epithelial detachment and lymphocytic infiltrates within the lamina propria and submucosa.

## Testing truth table of AND logic NPs

We first investigated each polymer construct for their stimuli response. For the pH-sensitive design, hydrolysis of the acyl hydrazone bond occurred only at pH <5.0, while negligible release was observed at pH >5.0, indicating that MSA-2 release requires severe acidic threshold (Supplementary Fig. 14a). To test the (pH–redox) sensitive PSC7A-Re-MSA-2 design, we chose 10 mM GSH to mimic the tumour reductive environment^27^. Data show that 10 mM GSH was able to induce MSA-2 release at either micelle (pH 7.4) or unimer state (pH 6.5, that is, regardless of the pH gates), illustrating the lack of AND logic for the dual stimuli response (Supplementary Fig. 14b).

**Fig. 2:**
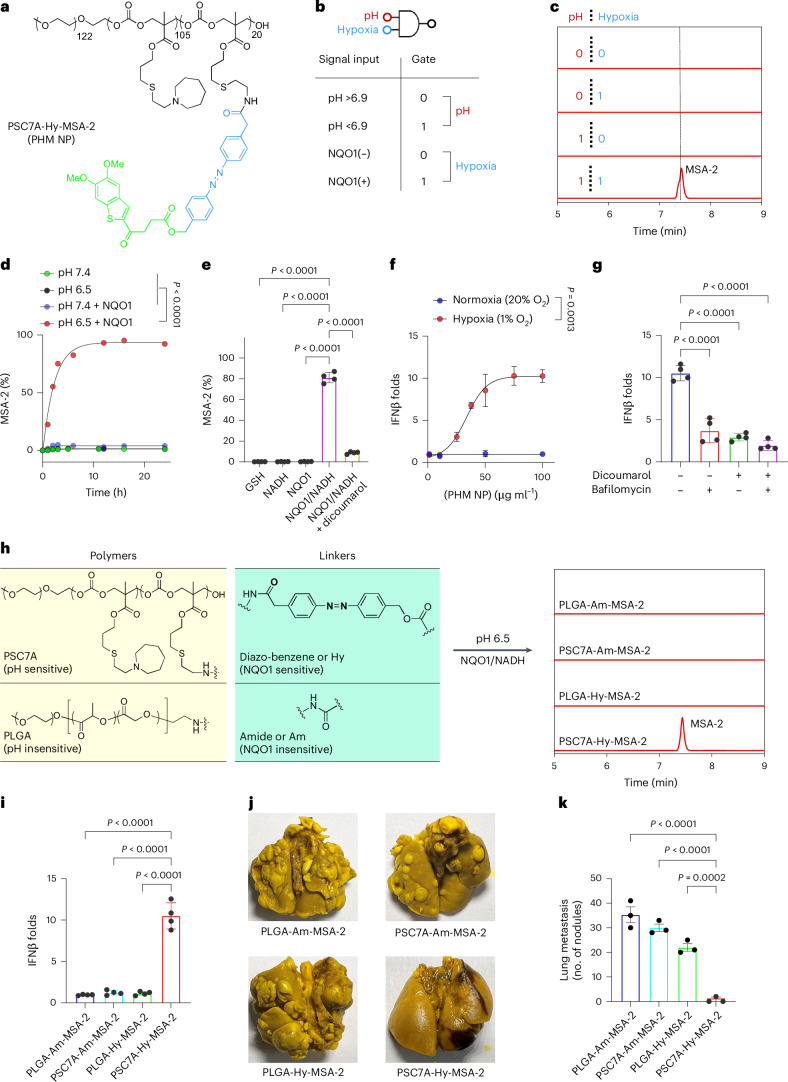
Evaluation of AND logic design for the STING-activating NPs. **a**, Chemical structure of the PSC7A-Hy-20MSA-2 (PHM) copolymer. **b**, pH–hypoxia AND logic gate for PHM NP. **c**, HPLC analysis of MSA-2 release from PHM NP (1 mg ml^−1^) incubated in PBS at 37 °C for 24 h, at pH 7.4 or 6.5, with or without NQO1 (100 µg ml^−1^)/NADH (800 µM). **d**, Kinetic analysis of MSA-2 release over 24 h for PHM NP (1 mg ml^−1^) under different environmental conditions. **e**, Release of MSA-2 from PHM NP (1 mg ml^−1^) at pH 6.5 under various incubation conditions: GSH (10 mM), NADH (800 µM), NQO1 (100 µg ml^−1^), NQO1 (100 µg ml^−1^)/NADH (800 µM) and NQO1 (100 µg ml^−1^)/NADH (800 µM)/dicoumarol (100 µM) (data represent the mean ± s.e.m., *n* = 4 tubes per group). **f**, Dose-dependent IFNβ secretion in THP1-ISG cells with PHM NP under normoxic (20% O_2_) or hypoxic (1% O_2_) conditions after 24 h incubation. **g**, IFNβ secretion in THP1-ISG cells co-incubated with PHM NP (50 µg ml^−1^) with or without dicoumarol (100 µM) or bafilomycin (250 nM) under hypoxic conditions (data represent the mean ± s.e.m., *n* = 3 wells per group). **h**, HPLC analysis of MSA-2 release for PLGA-Am-MSA-2 (4 mg ml^−1^), PSC7A-Am-MSA-2 (1 mg ml^−1^), PLGA-Hy-MSA-2 (4 mg ml^−1^) and PSC7A-Hy-MSA-2 (1 mg ml^−1^) incubated under pH 6.5 and NQO1 (100 µg ml^−1^)/NADH (800 µM) conditions. **i**, IFNβ secretion in THP1-ISG cells incubated with PLGA-Am-MSA-2 (0.4 mg ml^−1^), PSC7A-Am-MSA-2 (0.1 mg ml^−1^), PLGA-Hy-MSA-2 (0.4 mg ml^−1^) and PSC7A-Hy-MSA-2 (0.4 mg ml^−1^) under hypoxic conditions (1% O_2_) (data represent the mean ± s.e.m., *n* = 4 wells per group). **j**, Lung images from the LL/2 lung metastasis model following treatment with PLGA-Am-MSA-2 (80 mg kg^−1^), PSC7A-Am-MSA-2 (20 mg kg^−1^), PLGA-Hy-MSA-2 (80 mg kg^−1^) and PSC7A-Hy-MSA-2 (20 mg kg^−1^). **k**, Lung metastasis nodule counts from the treatment in **j** (data represent the mean ± s.e.m., *n* = 3 mice per group). The dosage of all polymer NPs was adjusted to equivalent MSA-2 dose across all formulations. *P* values were determined by ordinary one-way ANOVA (**e**,**g**,**i**,**k**) and by two-way ANOVA (**d**,**f**). Source data

For PSC7A-Hy-20MSA-2 (referred to as PHM NP; Fig. 2a), we investigated the (pH–hypoxia) AND logic design in drug release, STING activation and antitumour efficacy. In test tube studies, we deployed pH 6.5 and NQO1/NADH (100 µg ml^−1^ and 800 µM) to represent acidic pH and hypoxia triggers, respectively (Fig. 2b). When both conditions were met, PHM NPs demonstrated efficient MSA-2 release by high-performance liquid chromatography (HPLC), reaching 82% after 6 h and 92% after 24 h (Fig. 2c,d). By contrast, negligible release occurred in the presence of only one or neither stimulus. Higher NQO1 concentrations in the medium increased the release rate, which plateaued at 100 µg ml^−1^ (Extended Data Fig. 1b,c). Dicoumarol (100 µM), an NQO1 inhibitor, significantly inhibited MSA-2 release (Fig. 2e), underscoring the NQO1 specificity.

To evaluate the downstream signals upon STING activation, we incubated PHM NPs with THP1-ISG cells under normoxic (20% O_2_) or hypoxic (1% O_2_) conditions. Under 1% O_2_, PHM NPs triggered a 10-fold increase in interferon-β (IFNβ) production compared with 20% O_2_ (Fig. 2f). Dicoumarol (10 µM) as well as bafilomycin (100 nM), a vacuolar H^+^ ATPase inhibitor suppressing the acidification of endosomes and lysosomes, significantly reduced STING activation (Fig. 2g), affirming the necessity of dual stimuli in the activation of PHM NPs.

To further evaluate AND logic design over single-gated or non-responsive NPs, we constructed four polymer variants using PEO-*b*-poly(glycolic-co-lactic acid) (PLGA) as a pH-insensitive polymer and amide (Am) bond as a hypoxia-insensitive linker: PLGA-Am-MSA-2 (response, pH OFF, hypoxia OFF), PSC7A-Am-MSA-2 (pH ON, hypoxia OFF), PLGA-Hy-MSA-2 (pH OFF, hypoxia ON) and PSC7A-Hy-MSA-2 (PHM NP, pH ON, hypoxia ON). Drug release (Fig. 2h), IFNβ expression in THP1-ISG cells (Fig. 2i) and retardation of LL/2 metastasis (Fig. 2j,k) all showed the superior outcomes by the PHM NP over single-gated or non-responsive NPs.

## Pharmacokinetics, tissue biodistribution and cell tropism

**Fig. 3:**
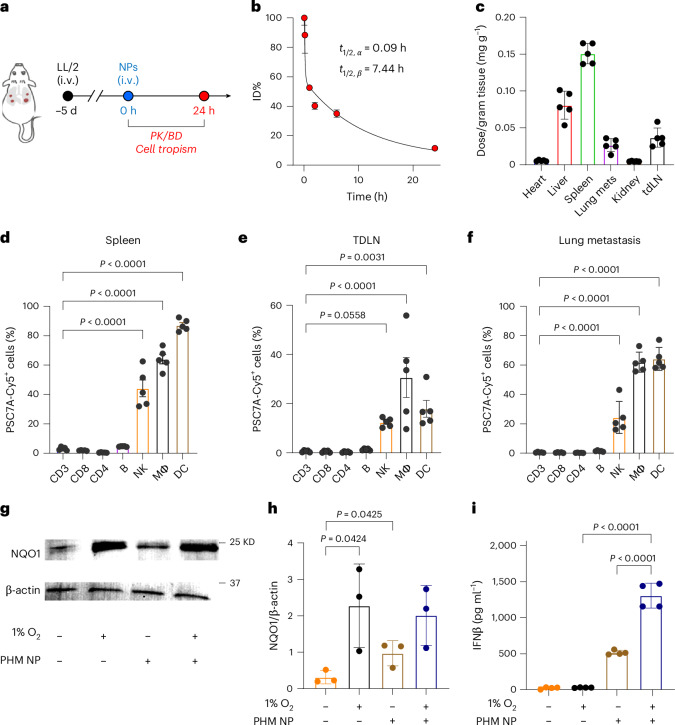
Analysis of pharmacokinetics, biodistribution and cell tropism of PHM NP. **a**, Plan of in vivo experiments. **b**, Pharmacokinetics of PSC7A-ICG/PHM mixed NPs (mass ratio 3:7) (20 mg kg^−1^) injected and analysed over 24 h (*n* = 5). **c**, Biodistribution in major organs at 24 h after i.v. injection of PSC7A-ICG/PHM mixed NPs (20 mg kg^−1^) (data represent the mean ± s.e.m., *n* = 5 mice per group). **d**–**f**, Cell tropism in the spleen (**d**), TDLN (**e**) and lung metastasis (**f**) at 24 h after injection of PSC7A-Cy5/PHM NP (20 mg kg^−1^) (data represent the mean ± s.e.m., *n* = 5 mice per group). **g**,**h**, Western blot (**g**) showing NQO1 expression in DC2.4 cells incubated with or without PHM NP (0.1 mg ml^−1^) under hypoxic or normoxic conditions with quantification data (**h**) (data represent the mean ± s.e.m., *n* = 3 independent experiments). **i**, IFNβ secretion from DC2.4 cells by ELISA assay (data represent the mean ± s.e.m., *n* = 4 wells per group). *P* values were determined by two-sided unpaired Student’s *t*-test (**h**) and ordinary one-way ANOVA (**d**–**f**,**i**). Source data

**Extended Data Fig. 4:**
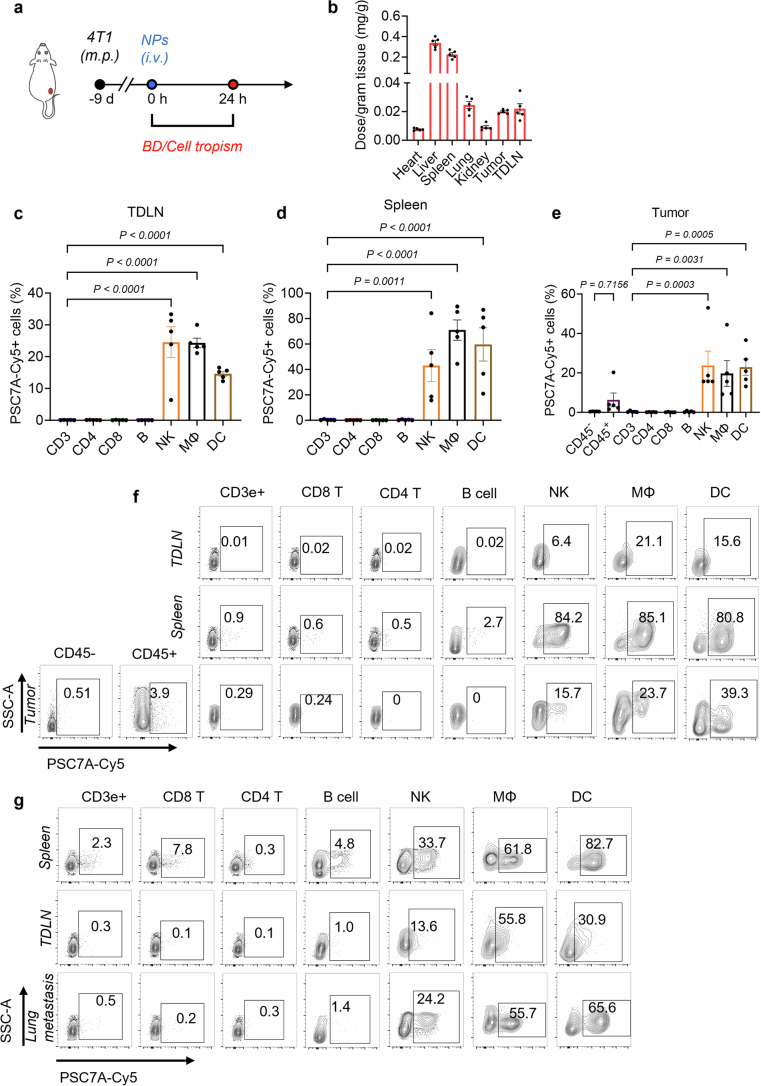
Biodistribution and cell tropism of PHM NP. **a**, Schematic of the *in vivo* experimental design. **b**, Biodistribution analysis of major organs 24 hours post-intravenous injection of mixed PSC7A-ICG/PHM NPs (weight ratio = 3:7, 20 mg/kg) in Balb/c mice bearing 4T1 breast tumors (n = 5 mice per group). **c-e**, Cellular tropism of PSC7A-Cy5/PHM NPs (weight ratio = 3:7, 20 mg/kg) in the TDLN, spleen and tumor 24 hours after administration (n = 5 mice per group). **f**, Representative dot plots of cell tropism in 4T1 tumor model post treatment (n = 5 mice per group). **g**, Representative dot plots of cell tropism in LL/2 tumor model post treatment. P values were determined by ordinary one-way ANOVA (**c**, **d** and **e**). Source data

We evaluated the stability of PHM NP in biological DMEM medium for 48 h (Supplementary Fig. 15a). The hydrodynamic diameter remained consistent at ~30 nm without signs of aggregation or precipitation. To evaluate the pharmacokinetics and biodistribution, we introduced a PSC7A polymer labelled with indocyanine green (ICG) into PHM NP (mass ratio PSC7A-ICG/PHM NP = 3:7) and administered them intravenously in the LL/2 model (Fig. 3a). Pharmacokinetic analysis revealed a biphasic clearance, with a short α-phase half-life of 0.09 h and an extended β-phase half-life of 7.44 h (Fig. 3b). Biodistribution analysis at 24 h post-injection showed preferential uptake in the spleen (0.15 ± 0.01 µg mg^−1^), followed by the liver (0.08 ± 0.02 µg mg^−1^), lymph nodes (0.04 ± 0.01 µg mg^−1^) and lungs (0.03 ± 0.009 µg mg^−1^) (Fig. 3c). Similar patterns were observed in the BALB/c 4T1 triple-negative breast orthotopic tumour model (Extended Data Fig. 4a,b). High accumulation of PHM NPs in the spleen facilitates immune surveillance against metastasis formation as circulating tumour cells often spread antigens through the blood for immune sensing^28–30^.

Cellular tropism was further examined by using Cy5-labelled PSC7A polymers in PHM NP. Across tissues examined in the LL/2 and 4T1 models, PHM NPs were predominantly taken up by CD45^+^ immune cells, with little uptake by CD45^−^ cancer cells in the 4T1 tumours (Extended Data Fig. 4c–f). Within the immune cell population, DCs, macrophages and NK cells are the major cell types that took up the NPs in the spleen, tumour-draining lymph nodes (TDLNs) and lung metastasis, whereas T and B cells exhibited negligible uptake across all examined tissues (Fig. 3d–f and Extended Data Fig. 4g). Pharmacological inhibition studies revealed that PHM NP uptake in DC2.4, RAW264.7 (macrophage) and NK-92 cells occurs primarily through clathrin-mediated endocytosis instead of caveolae/lipid raft-dependent internalization or macropinocytosis processes (Supplementary Fig. 15b–e). The selective accumulation in the antigen-presenting cells (APCs) highlights the tropic nature of PHM NPs within the tumour and lymphoid compartments.

Using DC2.4 cells as a model system, we found that hypoxic conditions (1% O_2_) significantly elevated NQO1 expression levels (Fig. 3g,h), but had no effect on IFNβ secretion (Fig. 3i). For further quantification, we used a methyl red assay^31^ and measured the NQO1 level that varied from 0.8 µg ml^−1^ to 80 µg ml^−1^ under normoxic to hypoxic conditions in DC2.4 cells, respectively (Extended Data Fig. 1d,e). Preincubation of PHM NP at higher NQO1 concentrations resulted in increased IFNβ secretion and STING activation (Extended Data Fig. 1f). Besides hypoxia, PHM NP treatment modestly elevated NQO1 levels under normoxic conditions, which led to moderate IFNβ secretion (0.5 ng ml^−1^). PHM NP treatment under hypoxia had comparable NQO1 levels to hypoxia-only control, but resulted in the highest level of IFNβ secretion (1.3 ng ml^−1^) from DC2.4 cells (Fig. 3i). We further analysed the constitutive and PHM NP-induced NQO1 levels in several cell lines. Data show that APCs and LL/2 cancer cells have constitutively higher activity of NQO1 than normal IMCD3 epithelial cells (Extended Data Fig. 1g).

## PHM NP efficacy depends on STING, cDC1 and CD8^+^ T cells

**Fig. 4:**
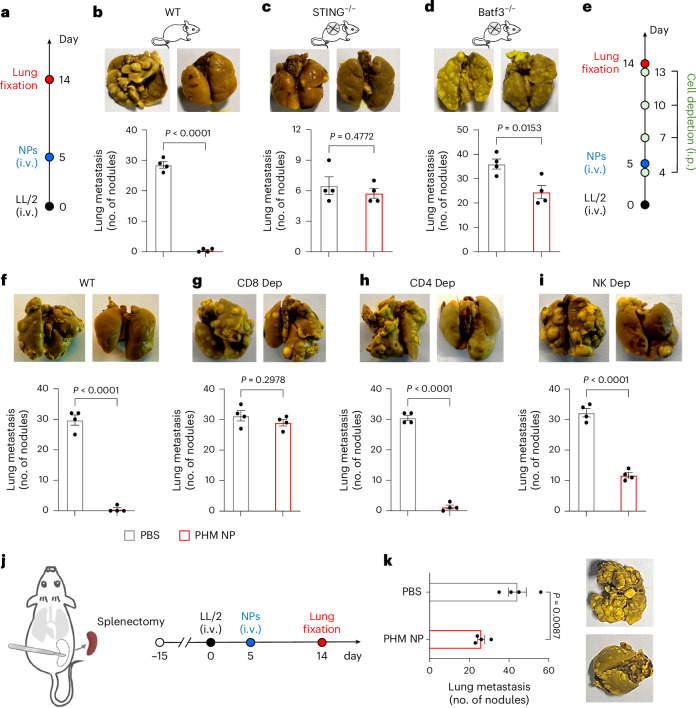
Mechanistic investigation of immune cell dependence of STING-mediated rejection of lung metastasis. **a**, Diagram of the experimental set-up. **b**–**d**, Lung nodule counts in WT (**b**), STING^−/−^ (**c**) and Batf3^−/−^ (**d**) mice following treatment with 20 mg kg^−1^ PHM NPs. **e**, Schematic of the immune cell depletion (Dep) experiment. **f**–**i**, Lung nodule counts in mice treated with 20 mg kg^−1^ PHM NP and no depletion (**f**), anti-CD8 antibody (200 μg per time) (**g**), anti-CD4 antibody (200 μg per time) (**h**) and anti-NK antibody (250 μg per time) (**i**). All antibodies were administered via intraperitoneal (i.p.) injection. **j**, Schematic of the splenectomy experiment. **k**, Lung nodule counts in mice treated with PBS or PHM NPs (20 mg kg^−1^). *P* values were determined by two-sided unpaired Student’s *t*-test (data represent the mean ± s.e.m., *n* = 4 mice per group). Source data

To elucidate the immune mechanism by PHM NPs, we evaluated their antitumour effect in genetically engineered murine models or antibody blocking of specific immune cell subset. In mice bearing LL/2 lung metastases, the absence of STING or cDC1 (*Tmem173*^*gt*^ and *Batf3*^−/−^ mice, respectively) markedly abrogated the therapeutic effect observed in wild-type (WT) controls (Fig. 4a–d). Notably, a reduction in lung nodule formation was observed in both treated and untreated groups of STING knockout mice. This observation can be attributed to the reduced activity of indoleamine 2,3-dioxygenase in STING-deficient mice, an enzyme whose absence impairs the development of LL/2 lung metastases^32^. These findings underscore the pivotal role of STING and cDC1 in driving PHM NP-mediated antitumour activity.

To further delineate the contributions of cytotoxic lymphocytes, we depleted NK cells, CD4^+^ and CD8^+^ T cells in tumour-bearing mice and assessed tumour progression following NP treatment (Fig. 4e–i). CD8^+^ T cell depletion resulted in a complete loss of antitumour efficacy (Fig. 4g, red curve, 29 ± 2.2 nodules), highlighting their essential role in tumour rejection. Partial abrogation of therapeutic effects was observed with NK cell depletion (Fig. 4i, red curve, 11.8 ± 1.7 nodules), indicating their auxiliary contribution. By contrast, CD4^+^ T cell depletion did not significantly impact metastasis suppression (Fig. 4h, red curve, 1.3 ± 1.3 nodules).

Next, we examined the contribution of spleen in PHM NP-mediated antitumour responses. LL/2 metastatic tumours were induced in splenectomized mice, and treatments were initiated 5 days post-inoculation (Fig. 4j). Without PHM NP treatment, splenectomized mice exhibited increased metastatic burden compared with WT controls (Fig. 4k versus 4f, grey curve, 44 ± 9 nodules versus 30 ± 3 nodules, *P* = 0.013, respectively). PHM NP treatment reduced lung nodule formation in splenectomized mice (Fig. 4k, 26 ± 4 versus 44 ± 9 nodules, respectively); however, the effect was less pronounced than that observed in WT mice (1.5 ± 1.0 nodules). These findings suggest that spleen plays an important role in endogenous and PHM NP-induced protection against metastasis formation in the LL/2 lung tumour model^28–30^.

**Fig. 5:**
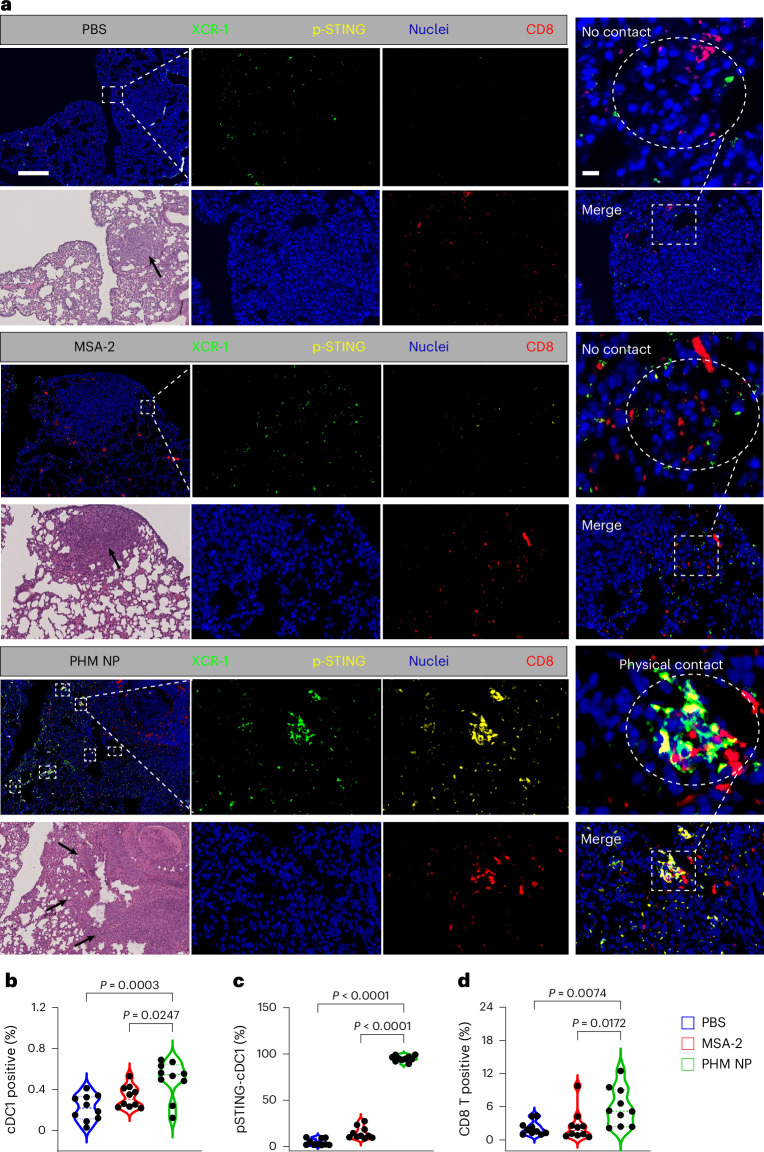
mIHC analysis reveals elevated cDC1-p-STING-CD8^+^ T cell signature after PHM NP therapy. **a**, Representative images of mIHC and haematoxylin and eosin (H&E) staining of LL/2 lung metastasis from PBS, MSA-2 (2.3 mg kg^−1^) and PHM NP (20 mg kg^−1^) treated mice (scale bars, 100 μm and 20 μm). The dose of MSA-2 matched that in the PHM NP. Black arrows indicate the tumour nodule. **b**, Quantification of the percentage of cDC1 cells in the LL/2 tumour nodules. **c**, Quantification of the percentage of p-STING^+^cDC1 cells within the total cDC1 population in the representative region. **d**, Quantification of the percentage of CD8^+^ T cells in the total immune cell population in the LL/2 tumour nodules (*n* = 10 metastasis areas). Box plots show median (centre line), 25th and 75th percentiles (box), and 5th and 95th percentiles (whiskers). *P* values were determined by ordinary one-way ANOVA (**b**–**d**) analysis. Source data

**Extended Data Fig. 5:**
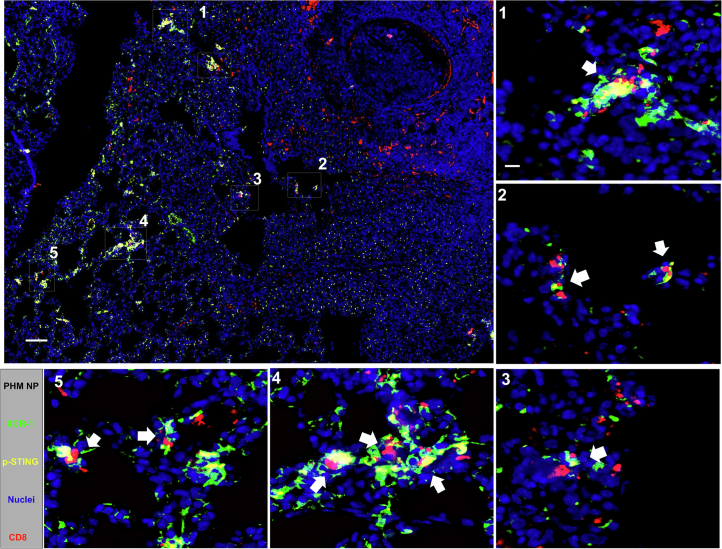
Detailed mIHC analysis of cDC1-p-STING-CD8^+^ T cell signature after PHM NP treatment. Representative images of mIHC staining of LL/2 lung metastasis from PHM NP (20 mg/kg) treated mice. Five representative regions are shown as indicated by the white boxes. White arrows highlight the physical contact region between cDC1 and CD8^+^ T cells (scale bar = 100 μm, 20 μm).

To investigate the spatial and functional dynamics of STING activation in the tumour microenvironment, we performed multiplex immunohistochemistry (mIHC) on formalin-fixed, paraffin-embedded LL/2 lung metastasis tissue. Using XCR1 (biomarker for cDC1), phospho-STING (activated STING) and CD8^+^ T (cytotoxic T cell marker) antibodies, we identified STING-activated cDC1 cells and their interactions with CD8^+^ T cells in the tumour regions (Fig. 5a and Extended Data Fig. 5). In the PHM NP-treated group, cDC1 cells in the lung nodules exhibited strong STING activation, evidenced by intense p-STING signals. Notably, direct physical interactions between p-STING^+^ cDC1 cells and CD8^+^ T cells occurred frequently in multiple metastatic foci (indicated by white boxes in Fig. 5a and Extended Data Fig. 5). Such interactions were largely absent in lung nodules from PBS or free MSA-2 treatment groups.

Quantitative image analysis shows that approximately 0.5% of the total immune cell population in the PHM NP-treated tumours were cDC1 cells, of which 95% were positive with p-STING signal (Fig. 5b,c). This is significantly higher than the 4.4% and 14.4% p-STING^+^cDC1 rates observed in the PBS and MSA-2 groups, respectively. In addition, the heightened cDC1 activation correlated with an increased recruitment of CD8^+^ T cells, which accounted for 5.9% of the immune cells in the tumour regions in the PHM NP group, markedly higher than the 2.1% and 2.6% observed in the PBS and MSA-2 groups, respectively (Fig. 5d). These results support the important role of STING activation in the priming of cDC1 cells, which subsequently recruit and engage CD8^+^ T cells to establish an effective antitumour immune response.

## Evaluation of PHM NP in metastatic cancer therapy

**Fig. 6:**
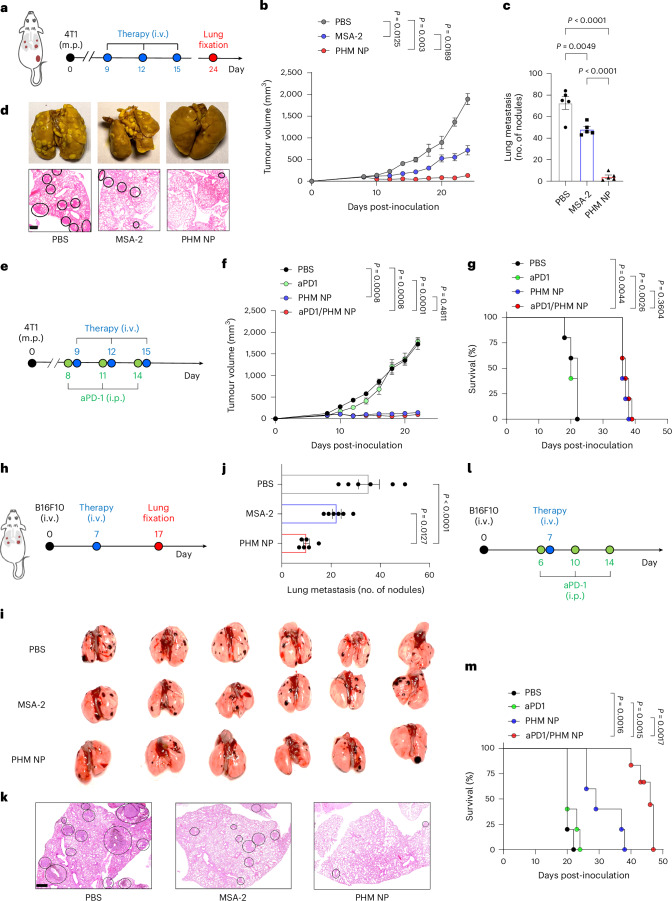
PHM NP significantly enhances the therapeutic effect in metastatic tumours. **a**, Schematic of the treatment plan in orthotopic 4T1 breast tumour and lung metastasis. **b**, Tumour growth curve of the primary 4T1 tumour after treatment with PBS, MSA-2 (1.2 mg kg^−1^, same dose with that in PHM NP) and PHM NP (10 mg kg^−1^). **c**,**d**, Lung metastasis nodule counts (**c**) and lung images and H&E images (**d**) on day 24 post-treatment (data represent the mean ± s.e.m., *n* = 5 mice per group; scale bar, 200 μm). **e**, Schematic of PHM NP with aPD1 treatments in mice bearing 4T1 tumours. **f**,**g**, Primary tumour growth curves (**f**) and survival outcomes (**g**) after treatment with PBS, aPD1, PHM NP and aPD1/PHM NP (*n* = 5 mice per group). **h**, Schematic of the treatment plan for B16F10 metastatic tumour. **i**–**k**, Lung metastasis nodule counts (**j**), lung images (**i**) and representative H&E staining images (**k**) on day 17 post-treatment of PBS, MSA-2 and PHM NP (data represent the mean ± s.e.m., *n* = 6 mice per group; scale bar, 200 μm). Black outlines in **d** and **k** delineate the tumour nodules. **l**, Schematic of PHM NP with immune checkpoint blockade treatments for B16F10 metastatic tumour. **m**, Survival curves after treatment with PBS, aPD1, PHM NP and aPD1/PHM NP (*n* = 5 mice per group). *P* values were determined by ordinary one-way ANOVA (**b**,**i**) analysis and two-way ANOVA with Bonferroni’s multiple-comparison tests (**c**,**f**) analysis, otherwise by the two-sided Mantel–Cox test (**g**,**m**). Source data

**Extended Data Fig. 6:**
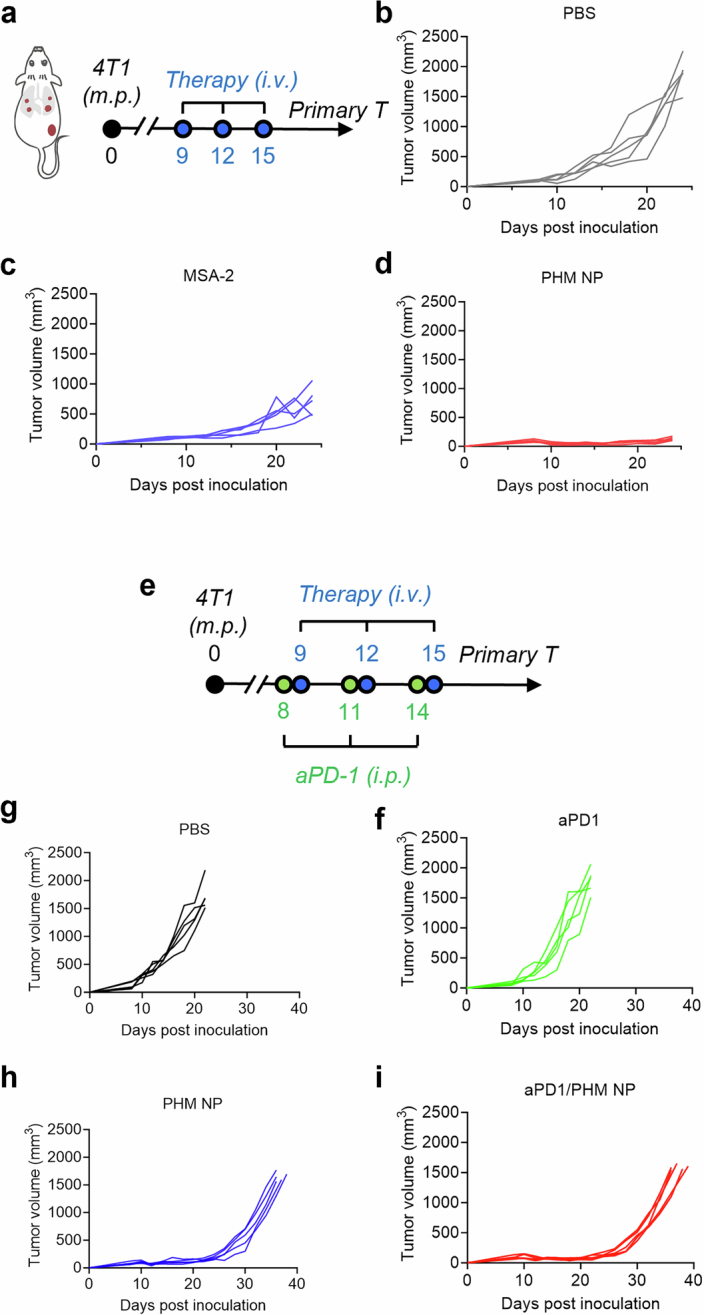
Individual primary tumor growth curves in 4T1 tumor bearing mice. **a**, Experimental diagram. **b-d**, Individual tumor growth curves from PBS, MSA-2 and PHM NP treatment (n = 5). **e**, Experimental scheme. **g-i**, Individual tumor growth curves under different treatments PBS, aPD1, PHM NP and aPD1/PHM NP respectively (n = 5).

To evaluate the therapeutic potential of PHM NPs across diverse metastatic cancer models, we investigated their effects in two additional settings: a 4T1 triple-negative breast cancer model and a B16F10 melanoma lung metastasis model. The 4T1 orthotopic model is characterized by spontaneous lung metastases by the second week post-inoculation. Intravenous administration of PHM NP (10 mg kg^−1^) was initiated when primary tumours in the mammary pad (m.p.) reached ~60–70 mm^3^, with injections given 3 times on days 9, 12 and 15 (Fig. 6a). Tumour growth and metastatic burden were evaluated on day 24. PHM NP significantly suppressed primary tumour growth compared with PBS and free MSA-2 groups (1.2 mg kg^−1^, equivalent dose to that in PHM NP; Fig. 6b and Extended Data Fig. 6a–d). Notably, systemic immune activation by PHM NP led to a dramatic reduction in lung metastasis, with counts reduced to 4.2 ± 1.6 nodules compared with 48 ± 2.4 for free MSA-2 and 72.6 ± 5.9 for PBS controls (Fig. 6c,d). PHM NP treatment also significantly improved survival rates relative to PBS and aPD1 monotherapy (Fig. 6e–g and Extended Data Fig. 6e–i). However, combining PHM NP with aPD1 did not yield additional benefits in primary tumour control or long-term survival in this model.

In the B16F10 melanoma model, one dose of PHM NP (10 mg kg^−1^) was administered on day 7 and lung metastatic nodules were assessed on day 17 (Fig. 6h). PHM NP treatment significantly reduced lung metastases to 10 ± 1.1 nodules, compared with 22.3 ± 1.8 for free MSA-2 and 35.3 ± 4.3 for PBS control (Fig. 6i,j). Histology analysis confirmed the reduced tumour burden in the lung parenchyma after PHM treatment over free MSA-2 and PBS controls (Fig. 6k). Monotherapy of PHM NP showed significantly improved survival outcome (median survival 30 days) than aPD1 or PBS control (20 days). Unlike the 4T1 model, combination therapy of PHM NP and aPD1 demonstrated a pronounced synergistic effect, achieving the longest survival (>50 days) over each arm of monotherapy (Fig. 6m). Overall, these findings suggest that PHM NP is effective in treating even immune-cold tumours such as 4T1 and B16F10^33,34^.

To further elucidate the cellular mechanism, we investigated DC/macrophage activation and T cell priming using a B16F10-ovalbumin (OVA) lung metastasis model. PHM NP treatment markedly increased the frequency of CD80^+^ and CD86^+^ DCs and macrophages over free MSA-2 and PBS controls (Supplementary Fig. 16). In the spleen, CD80^+^ and CD86^+^ DCs accounted for 59% and 78% of the total DC populations, respectively, compared with 15% and 12% in the MSA-2 group. In the B16F10-OVA tumours, CD80^+^ DCs reached 53% and CD86^+^ DCs 28% after PHM NP treatment versus 29% and 14% with MSA-2. The macrophages are also significantly polarized towards the M1 axis in the spleen and TDLN. For T cell priming, PHM NP treatment led to significant elevation of OVA-specific CD8^+^ T cell formation in the spleen over either MSA-2 or PBS controls (Supplementary Fig. 17). Notably, OVA-specific CD8^+^ T cells constituted 35% of the intratumoural CD8^+^ T cell population in the PHM NP group versus only 2% with MSA-2. In addition, PHM NP strongly promoted cytotoxic T cell activation (Supplementary Fig 18). In the spleen, GrzmB^+^ and IFNγ^+^ CD8^+^ T cells reached 72% and 18%, respectively, compared with 5% and 8% in the MSA-2 group. In the B16F10-OVA tumours, GrzmB^+^ CD8^+^ T cells and IFNγ^+^ CD8^+^ T cells reached 99% and 40% after PHM NP treatment, in contrast to 11% and 12% with MSA-2.

To evaluate long-term memory response, we analysed the formation of CD44^+^CD62L^−^ effector memory T cells (T_EM_). Data show that PHM NP induced a markedly higher proportion of memory CD8^+^ T cells—54% in the spleen and 78% in the tumours compared with 20% and 9%, respectively, in the MSA-2 group (Supplementary Fig. 19a–c). In the tumour rechallenge experiment, we utilized mice that survived 42 days (5 out of 8) after PHM NP treatment and re-inoculated the mice with LL/2 cells. Notably, 3 out of 5 mice were still alive on day 40 after rechallenge, whereas all naive control mice succumbed to tumour burden by day 18 (Supplementary Fig. 19d,e). Together, these findings provide direct evidence that PHM NP elicits long-term immunological memory and durable protection against tumour rechallenge.

## Discussion

Metastatic disease remains a formidable challenge in cancer therapy, with poor outcomes from conventional treatment modalities. STING signalling plays a pivotal role in antitumour immunity but presents a paradoxical duality in immune activation. On one hand, STING activation enhances immune surveillance, as evidenced by recent findings that STING activation in cancer cells suppresses metastatic outbreaks in lung adenocarcinoma^35^. On the other hand, chronic STING activation was shown to promote tumour progression through inflammation^36,37^. The contradictory roles complicate the therapeutic targeting of STING, necessitating precise control of their activation in selected cell populations and duration. Early clinical trials of small-molecule STING agonists (for example, Adu-S100 and MK-1454) have shown moderate clinical response but significant patient morbidities (grade 3/4 adverse effects)^11,12^. Poor tumour retention, lack of cell selectivity and STING-mediated T cell deaths are the recognized challenges^12,38,39^. Current STING-activating NPs offer advantages such as improved drug stability and tumour accumulation^30,40,41^. However, these systems often rely on single tumour stimulus to trigger drug release. This approach is limited owing to the analogue, overlapping signals in normal tissues, which can lead to on-target, off-tumour activity and systemic toxicity^42^. Intratumoural injection of STING-activating NPs can achieve strong local immune activation, but it may lack systemic efficacy^10,43^, while long-circulating formulations often raise safety concerns from broad immune stimulation^44^.

The molecular design of the AND logic NPs is rooted in the exploitation of tumour microenvironmental signals for STING-targeted immune activation. We combined MSA-2, a small-molecule STING agonist, with a non-canonical STING-activating PSC7A polymer, to achieve a synergistic immune activation via separate binding sites of the STING protein^22,43^. The use of an acyl hydrazone linker for MSA-2 conjugation requires severe acidic pH (<5.0) for release, whereas a disulfide linker revealed cleavage of MSA-2 from the PSC7A polymer even in the micelle state at pH 7.4 (Supplementary Fig. 14). The PHM NP releases MSA-2 only when both acidic pH and NQO1 signals are present (Fig. 2), allowing an AND logic design of STING agonist. Hypoxia is a hallmark of tumour microenvironmental signals, providing relevant stimulus for the hypoxia-dependent MSA-2 release^45^. Selected cell tropism towards APCs but not T cells (Fig. 3d–f) adds further benefit at driving STING signalling in cDC1 cells while avoiding STING-mediated T cell deaths (Fig. 5). Besides tumour uptake, PHM NPs also selectively accumulate in APCs in the spleen, where engagement of splenic DCs and macrophages primes antigen-specific cytotoxic T cell response with memory effect (Supplementary Figs. 16–19).

In conclusion, we present the conceptual design and preclinical validation of a (pH–hypoxia) AND-gated NPs to exploit STING signalling pathway, an important natural defence mechanism against immune evasive cancers. One potential limitation of the current therapy may reside in the treatment of early-stage or benign tumours, which may lack circulating tumour antigens for DC activation. In this case, tumour-specific protein or peptide antigens can be introduced in the PHM NP for direct priming of T cells. Nevertheless, the AND logic design represents a new paradigm in designing stimuli-responsive nanomedicine to achieve precise biological intervention, which may be adoptable for other therapeutic targets besides STING.

## Methods

### Preparation of AND logic NPs

PEG-*b*-P(SC7A-thioethylamine·HCl) was synthesized following the established protocols^21^. Different stimuli-responsive polymers were synthesized as described in Supplementary Figs. 1–3. To prepare NPs from each copolymer, 5 mg of the copolymer was first dissolved in 1 ml of PBS buffer at pH 5.0. Sodium hydroxide (0.1 M) was then added dropwise under continuous stirring until the pH reached 7.4, allowing micelle formation. The resulting NPs were characterized using dynamic light scattering (Zetasizer, Malvern Instruments) equipped with a He–Ne laser (*λ* = 633 nm, where *λ* is wavelength of the laser light).

### In vitro drug release test

PHM NP solutions were incubated at 37 °C for 24 h in PBS at pH 6.5 or pH 7.4, with or without NQO1/NADH. The release of MSA-2 was quantified using HPLC with a C18 column. The mobile phase consisted of solvent A as water with 0.08% trifluoroacetic acid and solvent B as acetonitrile with 0.08% trifluoroacetic acid. In addition, PHM NP solutions at pH 6.5 were incubated with various conditions, including GSH, NADH, NQO1, NQO1/NADH or NQO1/NADH with dicoumarol (NQO1 inhibitor), at 37 °C for 24 h.

### Cell line

THP1-Lucia ISG cells were purchased from InvivoGen (catalogue number thpl-isg). B16F10 cells were purchased from ATCC (catalogue number CRL-6475). 4T1 cells were provided by S. Huang, Massey Cancer Center, Virginia Commonwealth University. LL/2 cells were provided by Z. ‘James’ Chen, University of Texas (UT) Southwestern Medical Center (UT Southwestern). DC2.4 cells were provided by D. J. Siegwart, UT Southwestern. Raw264.7 cells were provided by Z. ‘James’ Chen. IMCD3 cells were provided by Y. Xun, UT Southwestern.

### Type I IFN reporter assay

THP1-Lucia ISG cells (InvivoGen) were seeded at 1 × 10^5^ cells per well in 96-well plates (180 µl per well) on day 1 and differentiated with phorbol 12-myristate 13-acetate (final 20–50 ng ml^−1^; 20 µl per well) for 3 h at 37 °C, 5% CO_2_. Cells were gently washed once with pre-warmed PBS and replenished with 200 µl pre-warmed complete growth medium (RPMI-1640 supplemented with 10% FBS and 1% penicillin–streptomycin, pH 7.4). On day 4, cells were washed once with pre-warmed PBS and 180 µl fresh medium was added, followed by 20 µl NP stock solution (final volume 200 µl). Plates were incubated for 24 h at 37 °C under hypoxia (1% O_2_, 5% CO_2_). Supernatants (10 µl per well) were transferred to white opaque 96-well plates, and QUANTI-Luc 4 reagent (InvivoGen) was prepared per the manufacturer’s instructions, loaded into the luminometer injector and dispensed for immediate luminescence measurement. Signal was recorded as relative light units (RLU), with background (medium-only) subtracted and data expressed as fold-induction over untreated control.

### Enzyme-linked immunosorbent assay

DC2.4 cells were seeded at a density of 5 × 10^5^ cells per well in 6-well plates and allowed to adhere overnight. Cells were then treated with PHM NP (100 µg ml^−1^) for the indicated time periods. At each time point, cell culture supernatants were collected, centrifuged to remove debris and analysed using the mouse CXCL10/IP-10/CRG-2 DuoSet enzyme-linked immunosorbent assay (ELISA) kit and the mouse IFNβ DuoSet ELISA kit (R&D Systems) according to the manufacturer’s instructions. The absorbance at 450 nm was measured using a microplate reader, and cytokine concentrations were calculated based on standard curves generated from recombinant mouse proteins.

### NQO1 activity assay

NQO1 enzymatic activity was quantified by monitoring the reduction of methyl red as a colorimetric substrate. In brief, methyl red (25 µg ml^−1^) and NADH (1 mM) were incubated with serial concentrations of recombinant mouse NQO1 protein (40 µg ml^−1^, 20 µg ml^−1^, 10 µg ml^−1^, 5 µg ml^−1^, 1 µg ml^−1^, 0.5 µg ml^−1^, 0.1 µg ml^−1^ and 0 µg ml^−1^) in 1× PBS at 37 °C. The decrease in absorbance at 430 nm was recorded using a microplate reader to generate a standard calibration curve. For intracellular quantification, DC2.4 cells were cultured under normoxic (20% O_2_) or hypoxic (1% O_2_) conditions for 24 h. Cell lysates were prepared from 3 × 10^5^ cells in 10 µl of lysis buffer, and intracellular NQO1 levels were determined based on the calibration curve.

### Micelle stability and cellular uptake mechanism

For micelle stability assessment, PHM NPs (1 mg ml^−1^) were dispersed in PBS (pH 7.4) or serum-containing medium and incubated at 37 °C. The particle size and zeta potential were monitored over time by dynamic light scattering to evaluate colloidal stability.

To investigate the cellular uptake mechanism, DC2.4 cells were pretreated with endocytosis inhibitors for 30 min before NP exposure: chlorpromazine (10 µg ml^−1^) to inhibit clathrin-mediated endocytosis by disrupting clathrin-coated pit formation; nystatin (50 µg ml^−1^) to block caveolae/lipid raft-dependent internalization by depleting membrane cholesterol; and amiloride (1 mM) to suppress macropinocytosis by inhibiting Na^+^/H^+^ exchange. Cells were then incubated with PHM NP/PSC7A-Cy5 (0.1 mg ml^−1^, mass ratio = 7:3) for the indicated period, washed and imaged using confocal laser scanning microscopy. The fluorescence intensity of internalized NPs was quantified by flow cytometry, and relative uptake among treatment groups was compared. Statistical significance was evaluated using one-way analysis of variance (ANOVA).

### Mice

STING^−/−^ and Batf3^−/−^ mice were purchased from the Jackson Laboratory, while C57BL/6 WT and BALB/c mice were obtained from Charles River Laboratories. All mice were kept under specific pathogen-free conditions in a barrier facility with a 12 h light–12 h dark cycle and fed standard chow (2916, Teklad Global). The experimental groups consisted of randomly selected female littermates, approximately 6–8 weeks old, of the same strain. All procedures were conducted in accordance with the ethical guidelines and protocols approved by the AAALAC-accredited Institutional Animal Care and Use Committee at UT Southwestern Medical Center under protocol number 2017-102331.

### Tumour models and treatment protocols

To establish the lung metastasis models, C57BL/6 mice were intravenously injected with either 1 × 10^6^ LL/2 lung cancer cells or 1 × 10^5^ B16F10 melanoma cells on day 0. The metastatic breast cancer model was generated in Balb/c mice by injecting 1 × 10^6^ 4T1 cancer cells into the mammary fat pad. For the LL/2 lung cancer model, tumour-bearing C57BL/6 mice were treated via i.v. injection of AND logic NPs (100 µl per mouse), MSA-2 or PBS (control) on day 5. Lungs were collected on day 14 and fixed in Bouin’s solution for 24 h. In the B16F10 melanoma model, mice were treated on day 7, and lungs were collected on day 17 following fixation in formalin for 24 h. For the 4T1 breast cancer model, mice were treated on days 9, 11 and 15. Primary tumour volumes were monitored, and lungs were collected on day 24 after fixation in Bouin’s solution for 24 h. In a subset of experiments, aPD1 (200 µg) was administered intraperitoneally 1 day before PHM NP treatment and repeated 3 times during the study. 4T1 tumour volumes were measured using calipers, with length (*L*) and width (*W*) recorded. Volumes were calculated using the formula *V* = *L* × *W* × *W*/2. According to the institutional animal care guidelines, the maximal permitted tumour size was 1,500 mm^3^, at which point animals were euthanized.

### Safety evaluations

Mouse serum was collected 6 h post-injection of PHM NP, MSA-2, PSC7A or PBS for cytokine analysis. The samples were analysed using the BD cytometric bead array (CBA) mouse inflammation kit (catalogue number 552364) and flow cytometry to quantify inflammatory cytokines. For liver and kidney function tests, serum samples were collected 24 h post-treatments. The samples were stored at 4 °C and subsequently sent to the UTSW Metabolic Phenotyping Core for analysis.

### Pharmacokinetic analysis

C57BL/6 mice bearing LL/2 lung metastases were intravenously administered dye-labelled PHM NP, formulated as a hybrid NP consisting of PHM NP and PSC7A-ICG at a mass ratio of 7:3. Each PSC7A polymer was conjugated with ~3 ICG molecules, and the total injected NP dose was 20 mg kg^−1^. At predefined time points post-injection, 50 µl of blood was collected from each mouse (*n* = 5 per group). Plasma was separated by centrifugation, diluted fivefold with PBS buffer containing 5 mM EDTA (pH 6.0), and fluorescence was quantified using the Pearl Trilogy Small Animal Imaging System (LI-COR). Fluorescent NPs were quantified at 800 nm (for ICG). Data were presented as the percentage of the injected dose (% ID), with plasma collected at 5 min post-injection representing the maximum dose (100% ID). Pharmacokinetic profiles were analysed using nonlinear regression and a two-phase decay model with GraphPad Prism software v10.5.0.

### Biodistribution analysis

To assess organ distribution, C57BL/6 mice bearing LL/2 lung metastases and Balb/c mice bearing 4T1 orthotopic solid tumours (~120 mm^3^) were intravenously injected with dye-labelled STING NPs as described above. Mice were euthanized 24 h post-injection, and tissues, including metastatic lung, TDLNs, liver, lungs, heart, kidney and spleen, were collected and weighed. Tissues were mechanically dissociated and homogenized in lysis buffer (2% Triton X-100, 100 mM HEPES, 5 mM EDTA, pH 7.1) using tissue grinder tubes (Precellys Lysing Kits). Homogenates were centrifuged at 500 × *g* for 3 min, and the supernatants were transferred to black 96-well plates for fluorescence quantification using the Pearl Trilogy Small Animal Imaging System (LI-COR). Fluorescent NPs were measured at 800 nm (ICG), and NP concentrations were calculated using tissue-specific standard curves generated from untreated mice. Organ uptake was reported as the percentage of injected dose per gram of tissue.

### Cell tropism studies

For cell tropism studies, C57BL/6 mice bearing LL/2 lung metastases and Balb/c mice bearing 4T1 orthotopic solid tumours (~120 mm^3^) were intravenously injected with Cy5-labelled STING NPs. Mice were euthanized 24 h post-injection, and tissues, including lung metastases, TDLNs and spleens, were collected and processed into single-cell suspensions. Cells were stained with fluorochrome-conjugated antibodies, including CD45 PerCP (clone 30-F11, BioLegend), MHC-II AF700 (clone M5/114.15.2, Invitrogen), CD11c BV605 (clone N418, BioLegend), CD11b PB (clone M1/70, BioLegend), CD4 FITC (clone RM4-5, BioLegend), CD8a PE (clone 53-6.7, BioLegend), CD3e BV786 (clone 145-2C11, BD Biosciences), F4/80 PE (clone BM8, Miltenyi Biotec), NK1.1 PE-Cy7 (clone PK136, BD Biosciences) and B220 APC-Cy7 (clone RA3-6B2, BioLegend). The LIVE/DEAD Fixable Aqua Dead Cell Stain Kit (Invitrogen, L34966) was used to assess cell viability. Data were acquired using BD LSRFortessa or Beckman CytoFLEX flow cytometers and analysed with CytExpert v2.4 and FlowJo v10.10.0. All gating strategies are shown in Supplementary Fig. 20.

### APC activation and T cell priming studies

For APC and T cell analysis, C57BL/6 mice bearing B16F10-OVA lung metastases were intravenously injected with PHM NPs at schematic time. Mice were euthanized 1 or 5 days post-injection, and tissues, including lung metastases, TDLNs and spleens, were collected and processed into single-cell suspensions. Cells were stained with fluorochrome-conjugated antibodies, including CD45 PerCP (clone 30-F11, BioLegend), MHC-II AF700 (clone M5/114.15.2, Invitrogen), CD11c BV605 (clone N418, BioLegend), CD11b PB (clone M1/70, BioLegend), F4/80 APC-Cy7 (clone BM8, BioLegend), CD80 PE-Cy7 (clone 16-10A1, BioLegend), CD86 APC (clone GL-1, BioLegend), CD206 PE (clone), CD4 PB (clone GK1.5, BioLegend), CD8a AF700 (clone QA17A07, BioLegend), CD3e BV786 (clone 145-2C11, BD Biosciences), H-2K^b^ OVA tetramer PE (SIINFEKL, MBL Life Sciences), CD62L PE-Cy7 (clone MEL-14, BioLegend), CD44 APC-Cy7 (clone IM7, BioLegend), GrzmB APC (clone QA16A02, BioLegend) and IFNγ PE (clone XMG1.2, BioLegend). The LIVE/DEAD Fixable Aqua Dead Cell Stain Kit (Invitrogen, L34966) was used to assess cell viability. Data were acquired using BD LSRFortessa or Beckman CytoFLEX flow cytometers and analysed with CytExpert v2.4 and FlowJo. All gating strategies are shown in Supplementary Figs. 20–23.

### mIHC analysis

Formalin-fixed, paraffin-embedded LL/2 lung tissues were collected for mIHC staining. mIHC was performed using the Opal 7-Color Manual IHC Kit (Akoya Biosciences, NEL811001KT) according to the manufacturer’s protocol. Multispectral images were acquired using the Akoya Biosciences Vectra Polaris system at ×20 magnification. For each slide, ten randomly selected fields were imaged. The resulting multispectral images were processed and analysed using the Halo v3.6.4134.464 image analysis software. Individual cells were identified using a nuclear segmentation algorithm based on DAPI staining, with a cellular mask applied around each nucleus. This allowed for the quantification of surface marker expression at the single-cell level. Staining agents are as follows: 4′,6-diamidino-2-phenylindole (DAPI; Thermo Fisher Scientific), phospho-STING (72971, Cell Signaling), anti-mouse/rat XCR1 antibody (148202, BioLegend) and CD8α (98941, Cell Signaling).

### Western blot

All reagents were obtained from Bio-Rad, and primary antibodies included anti-NQO1 (N5288, Sigma, 1:800) and β-actin (A2228, Sigma-Aldrich, mouse monoclonal antibody, 1:5,000). Cells were lysed in SDS sample buffer containing protease and phosphatase inhibitors, followed by heating for protein denaturation. The lysates were centrifuged, and the supernatant was loaded onto a 4–15% Mini-PROTEAN gel (Bio-Rad). Electrophoresis was performed at 50 V for 20 min, followed by 100 V for 60 min. Proteins were transferred to a PVDF membrane using 100 V for 60 min on ice. After transfer, membranes were blocked for 1 h at room temperature in either 5% non-fat milk or BSA (for phosphorylated proteins). Membranes were then incubated overnight at 4 °C with primary antibodies. Secondary antibodies (goat anti-mouse or goat anti-rabbit IgG, HRP-linked; BioLegend, 1:3,000) were applied for 1 h at room temperature. Protein bands were visualized using the GelDoc Go Gel Imaging System (Bio-Rad).

### In vivo immune cell depletion experiments

For NK cell depletion, mice were intraperitoneally injected with 500 μg of anti-NK1.1 antibody (clone PK136, BioXcell) on day 4 following LL/2 inoculation. Maintenance doses of 250 μg of anti-NK1.1 antibody were administered every 3 days. For CD8^+^ or CD4^+^ T cell depletion, mice received 200 μg of anti-CD8a antibody (clone YTS169.4, BioXcell) or anti-CD4 antibody (clone GK1.5, BioXcell) by intraperitoneal injection on day 4 after LL/2 cell injection. Maintenance doses of the same antibodies (200 μg) were administered every 3 days.

### Statistical analyses

Statistical analyses were conducted using Microsoft Excel and GraphPad Prism (version 9.0). Data are presented as the mean ± standard error of the mean (s.e.m.), unless otherwise specified. Appropriate post hoc statistical tests were applied throughout. For normally distributed datasets, one-way ANOVA followed by Tukey’s or Bonferroni’s multiple-comparison tests was used for comparisons among three or more groups, and two-way ANOVA with Bonferroni’s post hoc correction or main-effect-only models was applied for longitudinal tumour growth curves. For pairwise comparisons, two-tailed unpaired Student’s *t*-tests were used. Survival analysis was conducted using the Mantel–Cox test.

### Reporting summary

Further information on research design is available in the Nature Portfolio Reporting Summary linked to this article.

## Online content

Any methods, additional references, Nature Portfolio reporting summaries, source data, extended data, supplementary information, acknowledgements, peer review information; details of author contributions and competing interests; and statements of data and code availability are available at 10.1038/s41565-026-02130-3.

## Supplementary information

Supplementary Information Supplementary Methods, Figs. 1–23 and References.

Reporting Summary

## Source data

Source Data Fig. 1 Statistical source data.

Source Data Fig. 2 Statistical source data.

Source Data Fig. 3 Statistical source data/western blots.

Source Data Fig. 4 Statistical source data.

Source Data Fig. 5 Statistical source data.

Source Data Fig. 6 Statistical source data.

Source Data Extended Data Fig. 1 Statistical source data.

Source Data Extended Data Fig. 2 Statistical source data.

Source Data Extended Data Fig. 3 Statistical source data.

Source Data Extended Data Fig. 4 Statistical source data.

## Data Availability

All data supporting the findings of this study have been included in the article and its Supplementary Information. Source data are provided with this paper.

## Extended data

**Extended Data Table 1:** Quantification of drug-to-polymer ratios (DPRs)^a^ by UV-Vis spectroscopy

	PSC7A-pH-mMSA-2			PSC7A-Re-mMSA-2			PSC7A-Hy-mMSA-2		
Theoretical chains (m)	3	10	20	3	10	20	3	10	20
Actual MSA-2 numbers (m)	4.1	10.3	23.4	2.7	11.5	22.3	3.6	10	20.8

^a^The DPR for each polymer was calculated based on the absorbance measurements using the standard curves in Supplementary Fig. 7.

**Extended Data Table 2:** Hydrodynamic diameter and polydispersity index (PDI)^a^ of NPs

	PSC7A-pH-mMSA-2			PSC7A-Re-mMSA-2			PSC7A-Hy-mMSA-2		
MSA-2 number	3	10	20	3	10	20	3	10	20
Size(nm)	29 ± 7	26 ± 7	29 ± 7	28 ± 7	28 ± 7	30 ± 7	29 ± 7	28 ± 7	28 ± 7
PDI	0.1	0.11	0.1	0.1	0.1	0.09	0.09	0.1	0.09

^a^The hydrodynamic diameter and PDI of all NPs formulations were measured by dynamic light scattering (DLS) (n = 3). Data are shown as mean ± standard error of the mean.
