# Supplementary material for: AND logic nanoparticle for precision immunotherapy of metastatic cancers
Source: Nat Nanotechnol. 2026 Mar 9;21(4):606–16. doi: 10.1038/s41565-026-02130-3 (PMC13106039; doi:10.1038/s41565-026-02130-3)
Supplement: Supplementary file 1 — Supplementary Methods, Figs. 1–23 and References. [file 41565_2026_2130_MOESM1_ESM.pdf]

nature nanotechnology

Supplementary information https://doi.org/10.1038/s41565-026-02130-3

AND logic nanoparticle for precision immunotherapy of metastatic cancers

In the format provided by the authors and unedited

<u>Table of Contents</u>

Supplementary Methods…………………………………………………………………...2

Supplementary Figures……………………………..…………………………………...8

Supplementary Reference………………………….…..…………………………….....31

Supplementary Method

Chemicals

All chemicals were purchased from Sigma-Aldrich unless otherwise described. NADH disodium salt, ICG-OSu and Cy5-NHS Ester were purchased from MedChemExpress. Human NQO1 (SRP6539) was purchased from Sigma-Aldrich. Dicoumarol was purchased from TCI. Bafilomycin A1 was purchased from RPI research product international. NH_2-PLGA(5k)-mPEG(5k) was purchased from Boardpharm.

Cell lines and culture

THP1-Lucia ISG cells, B16F10 cells were purchased from American Type Culture Collection (ATCC, Manassas, VA). 4T1 cells were provided by S. Huang, Massey Cancer Center, Virginia Commonwealth University. LL/2 were provided by Z. J. Chen, University of Texas Southwestern Medical Center (UT Southwestern). DC 2.4 cells were provided by D. J. Siegwart, UT Southwestern. All the cells were cultured in complete Dulbecco's modified Eagle's medium (Gibco BRL Life Technologies, Canada) supplemented with 10% fetal bovine serum (FBS) (HyClone, USA), penicillin (100 U/ml), and streptomycin (100 mg/ml; Gibco-BRL Life Technologies, Canada). THP1-Lucia ISG cells were cultured in RPMI medium supplemented with 10% FBS. All cells were grown at 37°C in a humid atmosphere with 5% CO_2. All cells used in this study tested negative for mycoplasma contamination.

Synthesis of tert-butyl 2-(3-mercaptopropanoyl)hydrazine-1-carboxylate (1-1)

In a solution containing 3-mercaptopropionic acid (1.0 g, 9.4 mmol) in 10 mL of dichloromethane (DCM), 1-(3-dimethylaminopropyl)-3-ethylcarbodiimide hydrochloride (EDCI, 3.6 g, 18.8 mmol), and N-hydroxysuccinimide (NHS, 1.3 g, 11.3 mmol) were introduced. The reaction mixture was stirred at room temperature for 1 h, followed by the addition of tert-butyl carbazate (1.5 g, 11.3 mmol), and stirring was continued at room temperature overnight. The resulting crude product was subjected to washing with CH_2Cl_2/water and drying over sodium sulfate. The final product was isolated by column chromatography on silica gel, yielding **1-1** (1 g,

50%). ^{1}H NMR (400 MHz, $CDCl_3$) δ 2.77 (t, 2H, $-CH_2-$), 2.55 (t, J = 6.8 Hz, 2H, $-CH_2-$), 1.43 (s, 9H, $-3(CH_3)_3$). LC-MS: $[M+H]^+$: 221.1.

Synthesis of PEG-*b*-pMAC-m(tert-butyl 2-(3-mercaptopropanoyl)hydrazine-1-carboxylate) (1-2)

PEG-*b*-pMAC (1 g) and **1-1** (m=3: 21.4 mg, m=10: 71.0 mg, m=20: 142 mg) were dissolved in 50 mL of dimethylformamide (DMF). Subsequently, 2,2-dimethoxy-2-phenylacetophenone (DMPA) (19.4 mg, 75.7 μmol) was added, and the reaction proceeded under UV light for 2 hours at room temperature. Following this, C7ASH HCl (12.3 g, 62.8 mmol) was introduced into the reaction solution, and the remaining DMPA (19.4 mg, 75.7 μmol) was added under UV light. The reaction further continued for 16 h at room temperature. The resulting crude product was subjected to dialysis in water, followed by lyophilization, yielding the final product **1-2**. ^{1}H NMR (400 MHz, CDCl3) δ 4.24 (d, J = 20.0 Hz, 6H, $-CH_2CCH_2-$, $-CH_2-$), 3.62 (s, 2H, $-OCH_2CH_2O-$), 3.35 (s, 3H, $-CH_3$), 3.24 (s, 6H, $-CH_2-$, $-CH_2NCH_2-$), 3.01 (s, 2H, $-SCH_2-$), 2.62 (d, J = 6.8 Hz, 2H, $-CH_2-$), 2.14 (s, 2H, $-CH_2-$), 1.94 (t, J = 7.0 Hz, 4H, $-CH_2-$, $-CH_2-$), 1.68 (d, J = 15.4 Hz, 4H, $-CH_2-$, $-CH_2-$), 1.41 (s, 9H, $-3(CH_3)_3$), 1.25 (d, J = 4.1 Hz, 3H, $-CH_3$).

Synthesis of PSC7A-pH-mMSA-2

1-2 (100 mg) and MSA-2 (m=3: 10 mg, m=10: 30 mg, m=20: 60 mg) were dissolved in 5 mL of DCM with 0.5 mL trifluoroacetic acid (TFA), and the reaction proceeded overnight. The resulting reaction mixture was then dialyzed with methanol to obtain the final product. ^{1}H NMR (400 MHz, CDCl3) δ 7.90 (s, 1H, Ar-H), 7.24 (s, 2H, Ar-H), 4.26 (d, J = 20.0 Hz, 6H, $-CH_2CCH_2-$, $-CH_2-$), 3.98 (d, J = 9.4 Hz, 6H, $-CH_3$), 3.64 (s, 2H, $-OCH_2CH_2O-$), 3.37 (s, 3H, $-CH_3$), 3.23 (s, 6H, $-CH_2-$, $-CH_2NCH_2-$), 3.02 (s, 2H, $-SCH_2-$), 2.63 (d, J = 6.8 Hz, 2H, $-CH_2-$), 2.16 (s, 2H, $-CH_2-$), 1.95 (t, J = 7.0 Hz, 4H, $-CH_2-$, $-CH_2-$), 1.68 (d, J = 15.4 Hz, 4H, $-CH_2-$, $-CH_2-$), 1.44 (s, 9H, $-3(CH_3)_3$), 1.26 (d, J = 4.1 Hz, 3H, $-CH_3$).

Synthesis of 2-(pyridin-2-yldisulfaneyl)ethan-1-ol (2-1)

1,2-Di(pyridin-2-yl)disulfane (5.6 g, 25.6 mmol) was dissolved in 50 mL of ethanol with stirring. Subsequently, 2-mercaptoethanol (1.0 g, 12.8 mmol) was slowly added dropwise to the solution, and the reaction proceeded for 6 h at rt. The solvent was then evaporated, and the resulting

mixture was washed with ethyl acetate and water. Afterward, the crude product was dried over sodium sulfate. Purification of the crude product was accomplished through silica gel column chromatography, resulting in **2-1** (1.5 g, 62.5%).^{1}H NMR (400 MHz, $CDCl_3$) δ 7.66 – 7.52 (m, 2H, Py-H), 7.39 (t, J = 7.9 Hz, 1H, Py-H), 6.77 (t, J = 6.7 Hz, 1H, Py-H), 3.91 (t, J = 5.8 Hz, 2H, -CH_2-), 2.88 (t, J = 5.8 Hz, 2H, -CH_2-). LC-MS: $[M+H]^+$: 188.0.

Synthesis of 2-(pyridin-2-yldisulfaneyl)ethyl 4-(5,6-dimethoxybenzo[b]thiophen-2-yl)-4-oxobutanoate (2-2)

MSA-2 (157 mg, 534 μmol) and **2-1** (100 mg, 534 μmol) were dissolved in 50 mL of CH_2Cl_2. Subsequently, 4-dimethylaminopyridine (DMAP) (13.1 mg, 107 μmol) and EDCI (204 mg, 1.07 mmol) were added to the solution, and the reaction proceeded for 6 h. The solvent was then evaporated, and the residue was washed with CH_2Cl_2/water. The crude product was further purified through a silica gel column, yielding **2-2** (195.2 mg, 78.9%). ^{1}H NMR (400 MHz, $CDCl_3$) δ 8.46 (ddd, J = 4.8, 1.8, 1.0 Hz, 1H, Py-H), 7.89 (d, J = 0.6 Hz, 1H, Ar-H), 7.68 (s, 1H, Py-H), 7.66 (dd, J = 7.1, 1.8 Hz, 1H, Py-H), 7.25 (d, J = 2.0 Hz, 2H, Ar-H), 7.09 (ddd, J = 7.1, 4.8, 1.4 Hz, 1H, Py-H), 4.37 (t, J = 6.4 Hz, 2H, -CH_2-), 3.96 (d, J = 10.0 Hz, 6H, -CH_3), 3.31 (t, J = 6.7 Hz, 2H, -CH_2-), 3.05 (t, J = 6.4 Hz, 2H, -CH_2-), 2.78 (t, J = 6.7 Hz, 2H, -CH_2-). LC-MS: $[M+H]^+$: 464.1.

Synthesis of 3-((2-((4-(5,6-dimethoxybenzo[b]thiophen-2-yl)-4-oxobutanoyl)oxy)ethyl)disulfaneyl)propanoic acid (2-3)

2-2 (100 mg, 216 μmol) was dissolved in a mixture of 50 mL CH_2Cl_2 and ethanol (EtOH). Subsequently, 3-mercaptopropionic acid (27.5 mg, 259 μmol) was added dropwise to the solution, and the reaction was allowed to proceed for 6 h. The solvent was then evaporated, and the resulting residue was washed with CH_2Cl_2 /water. The crude product underwent further purification through a silica gel column, affording **2-3** (79.1 mg, 80% yield). ^{1}H NMR (400 MHz, CDCl3) δ 7.89 (s, 1H, Ar-H), 7.25 (s, 2H, Ar-H), 4.37 (t, J = 6.6 Hz, 2H, -CH_2-), 3.96 (d, J = 9.6 Hz, 6H, -CH_3), 3.32 (d, J = 6.7 Hz, 2H, -CH_2-), 2.99 – 2.88 (m, 4H, -CH_2-), 2.86 – 2.75 (m, 4H, -CH_2-). LC-MS: $[M+H]^+$: 458.1.

Synthesis of 2-((3-((2,5-dioxopyrrolidin-1-yl)oxy)-3-oxopropyl)disulfaneyl)ethyl 4-(5,6-dimethoxybenzo[b]thiophen-2-yl)-4-oxobutanoate (2-4)

2-3 (50.0 mg, 109 μmol) and EDCI (41.7 mg, 218 μmol) were dissolved in 50 mL of CH_2Cl_2. Subsequently, NHS (15.1 mg, 131 μmol) was added to the solution, and the reaction proceeded for 2 h. The solvent was then evaporated, and the resulting residue was washed with CH_2Cl_2/water, yielding **2-4** (50.1 mg, 82.5%). ^{1}H NMR (400 MHz, CDCl3) δ 7.89 (s, 1H, Ar-H), 7.25 (s, 2H, Ar-H), 4.37 (t, J = 6.4 Hz, 2H, -CH_2-), 3.96 (d, J = 9.4 Hz, 6H, -CH_3), 3.32 (t, J = 6.7 Hz, 2H, -CH_2-), 3.09 – 3.03 (m, 2H, -CH_2-), 3.03 – 2.97 (m, 2H, -CH_2-), 2.94 (t, J = 6.5 Hz, 2H, -CH_2-), 2.88 – 2.71 (m, 6H, -CH_3), 1.33 – 1.20 (m, 2H, -CH_2-). LC-MS: $[M+H]^+$: 556.1.

Synthesis of PSC7A-Re-mMSA-2

PSC7A-mNH2 (100 mg) and **2-4** (m=3: 10 mg, m=10: 30 mg, m=20: 60 mg) were dissolved in 5 mL of dimethylformamide (DMF), then K_2CO_3 (5 mg) was added to the solution, and the reaction proceeded overnight. The reaction mixture was then dialyzed with methanol to obtain the final product. ^{1}H NMR (400 MHz, $CDCl_3$) δ 7.89 (s, 1H, Ar-H), 7.25 (s, 2H, Ar-H), 4.21 (d, J = 20.0 Hz, 6H, -CH_2CCH_2-, -CH_2-), 3.96 (d, J = 9.4 Hz, 6H, -CH_3), 3.59 (s, 2H, -OCH_2CH_2O-), 3.33 (s, 3H, -CH_3), 3.27 – 3.04 (s, 2H, -SCH_2-), 2.62 (d, J = 6.8 Hz, 2H, -CH_2-), 2.14 (s, 2H, -CH_2-), 1.94 (t, J = 7.0 Hz, 4H, -CH_2-, -CH_2-), 1.68 (d, J = 15.4 Hz, 4H, -CH_2CH_2-), 1.25 (d, J = 4.1 Hz, 3H, -CH_3).

Synthesis of tert-butyl 2-(4-nitrosophenyl)acetate (3-1)

Tert-butyl 2-(4-aminophenyl)acetate (1 g, 4.8 mmol) was dissolved in 50 mL of CH_2Cl_2 and added to a solution of Oxone® (3.0 g, 7.7 mmol) in water. Subsequently, the reaction mixture was stirred for 4.5 hours at room temperature. Afterward, the deep green organic layer was separated, and the aqueous solution was neutralized by the addition of a saturated solution of $NaHCO_3$ (50 mL). The mixture was then washed with CH_2Cl_2/water, dried over Na_2SO_4, resulting in the product of **3-1**. ^{1}H NMR (400 MHz, CDCl3) δ 8.18 (d, 2H, J = 8.7 , Ar-H), 7.44 (d, 2H, J = 8.7, Ar-H), 3.63 (s, 2H, Ar-CH_2), 1.44 (s, 9H, -$(CH_3)_3$). LC-MS: $[M+H]^+$: 222.1.

Synthesis of tert-butyl 2-(4-((4-(hydroxymethyl)phenyl)diazenyl)phenyl)acetate (3-2)

3-1 (500 mg, 2.71 mmol) was dissolved in 10 mL of ethanol containing acetic acid (AcOH, 0.5 mL) with stirring at 0 °C. Subsequently, 4-aminobenzyl alcohol (334 mg, 2.71 mmol) was added slowly to the solution, and the reaction proceeded for 2 h at room temperature. The solvent

was then evaporated, and the resulting residue was washed with CH_2Cl_2/water. The crude product underwent further purification through a silica gel column, yielding **3-2** (650 mg, 88.1%). ^{1}H NMR (400 MHz, $CDCl_3$) δ 8.25 (d, J = 8.6 Hz, 2H, Ar-H), 8.16 (d, J = 8.6 Hz, 2H, Ar-H), 7.42 – 7.35 (m, 4H, Ar-H), 3.61 (s, 2H, $ArCH_2$-), 3.58 (s, 2H, $ArCH_2$-), 1.44 (s, 9H, -$(CH_3)_3$). LC-MS: $[M+H]^+$: 327.2.

Synthesis of 4-((4-(2-(tert-butoxy)-2-oxoethyl)phenyl)diazenyl)benzyl 4-(5,6-dimethoxybenzo[b]thiophen-2-yl)-4-oxobutanoate (3-3)

MSA-2 (50.0 mg, 170.0 µmol) and **3-2** (66.5 mg, 203.9 µmol) were dissolved in 15 mL of CH_2Cl_2. Subsequently, DMAP (4.2 mg, 34.0 µmol) and EDCI (65.0 mg, 340.0 µmol) were added to the solution, and the reaction proceeded for 6 h at room temperature. The solvent was then evaporated, and the resulting residue was washed with CH_2Cl_2/water. The crude product was further purified through a silica gel column, yielding **3-3** (90.0 mg, 87.9%). ^{1}H NMR (400 MHz, CDCl3) δ 7.94 – 7.78 (m, 5H, Ar-H), 7.48 (d, J = 8.4 Hz, 2H, Ar-H), 7.43 (d, J = 8.4 Hz, 2H, Ar-H), 7.24 (d, J = 6.0 Hz, 2H, Ar-H), 5.22 (s, 2H, $ArCH_2$-), 3.96 (d, J = 9.7 Hz, 6H, -CH_3), 3.61 (s, 2H $ArCH_2$-), 3.35 (t, J = 6.7 Hz, 2H, -CH_2-), 2.88 (t, J = 6.7 Hz, 2H, -CH_2-), 1.45 (s, 9H, -$(CH_3)_3$). LC-MS: $[M+H]^+$: 603.2.

Synthesis of 4-((4-(2-((2,5-dioxopyrrolidin-1-yl)oxy)-2-oxoethyl)phenyl)diazenyl)benzyl 4-(5,6-dimethoxybenzo[b]thiophen-2-yl)-4-oxobutanoate (3-4)

3-3 (50.0 mg, 83.0 µmol) was dissolved in 10 mL of CH_2Cl_2, then 10 mL of trifluoroacetic acid (TFA) was added with further stirring for 15 mins at 0 °C. Subsequently, the solvent was evaporated, and the resulting residue was dissolved in 10 mL of CH_2Cl_2 without requiring further purification, proceeding directly to the next step. EDCI (31.7 mg, 166.0 µmol) and NHS (11.5 mg, 99.6 µmol) were added to the solution, and the reaction proceeded for 2 h at rt. The solvent was then evaporated, and the resulting residue was washed with CH_2Cl_2/water, yielding **3-4** (50.0 mg, 93.6%). ^{1}H NMR (400 MHz, $CDCl_3$) δ 8.02 – 7.81 (m, 5H, Ar-H), 7.49 (dq, J = 11.0, 5.9, 4.1 Hz, 4H, Ar-H), 7.23 (d, J = 5.6 Hz, 2H, Ar-H), 5.21 (s, 2H, $ArCH_2$-), 4.11 (q, J = 7.2 Hz, 4H, -CH_2-), 4.01 (s, 2H, $ArCH_2$-), 3.96 (d, J = 9.7 Hz, 6H, -CH_3), 3.95 (dd, J = 10.2, 2.9 Hz, 4H, -CH_2-). LC-MS: $[M+H]^+$: 644.2.

Synthesis of PSC7A-Hy-mMSA-2

PSC7A-mNH2 (100 mg) and **3-4** (m=3: 10 mg, m=10: 30 mg, m=20: 60 mg) were dissolved in 5 mL of dimethylformamide (DMF), then K_2CO_3 (5 mg) was added to the solution, and the reaction proceeded overnight at rt. The reaction mixture was then dialyzed with methanol to obtain the final product. ^{1}H NMR (400 MHz, $CDCl_3$) δ 8.02 – 7.81 (m, 5H, Ar-H), 7.49 (dq, J = 11.0, 5.9, 4.1 Hz, 4H, Ar-H), 7.23 (d, J = 5.6 Hz, 2H, Ar-H), 5.21 (s, 2H, $ArCH_2$-), 4.24 (d, J = 20.0 Hz, 6H, -CH_2CCH_2-, -CH_2-), 3.96 (d, J = 9.4 Hz, 6H, -CH_3), 3.62 (s, 2H, -OCH_2CH_2O-), 3.35 (s, 3H, -CH_3), 3.24 (s, 6H, -CH_2-, -CH_2NCH_2-), 3.01 (s, 2H, -SCH_2-), 2.62 (d, J = 6.8 Hz, 2H, -CH_2-), 2.14 (s, 2H, -CH_2-), 1.94 (t, J = 7.0 Hz, 4H, -CH_2-, -CH_2-), 1.68 (d, J = 15.4 Hz, 4H, -CH_2-, -CH_2-), 1.25 (d, J = 4.1 Hz, 3H, -CH_3).

Supplementary Figures

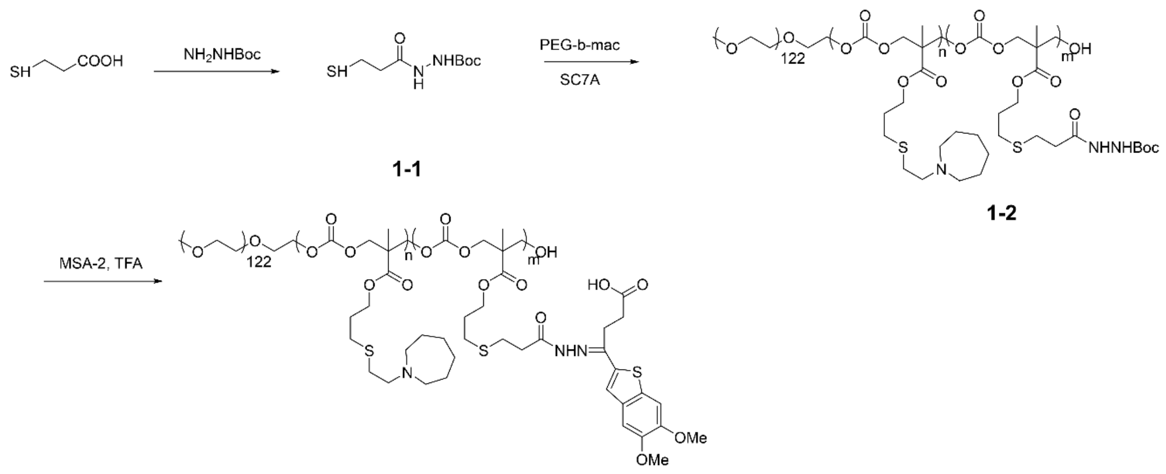

Supplementary Figure 1. Schematic of synthesis of PEO-*b*-PSC7A-pH-mMSA-2 copolymer.

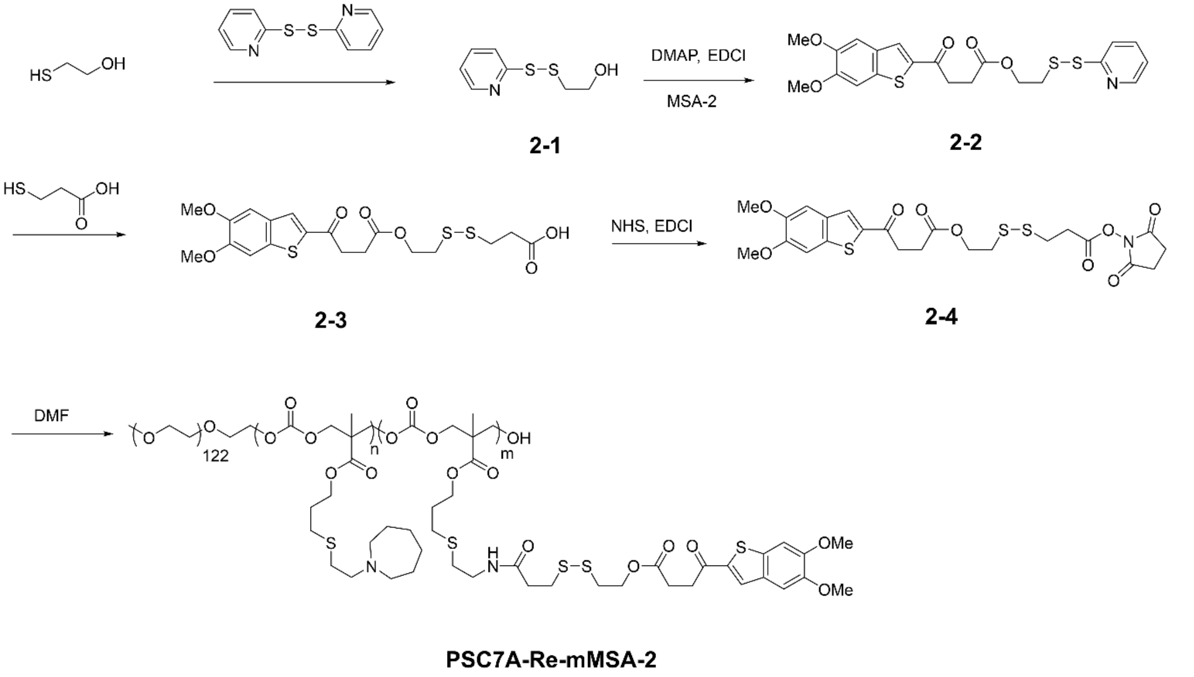

Supplementary Figure 2. Schematic of synthesis of PEO-*b*-PSC7A-Re-mMSA-2 copolymer.

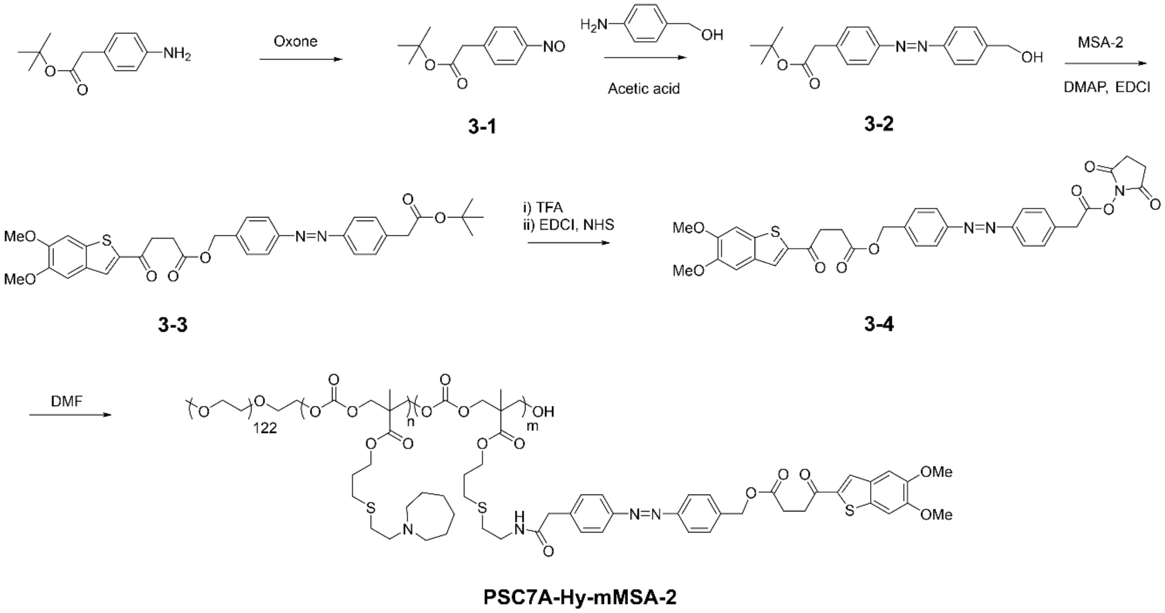

Supplementary Figure 3. Schematic of synthesis of PEO-*b*-PSC7A-Hy-mMSA-2 copolymer.

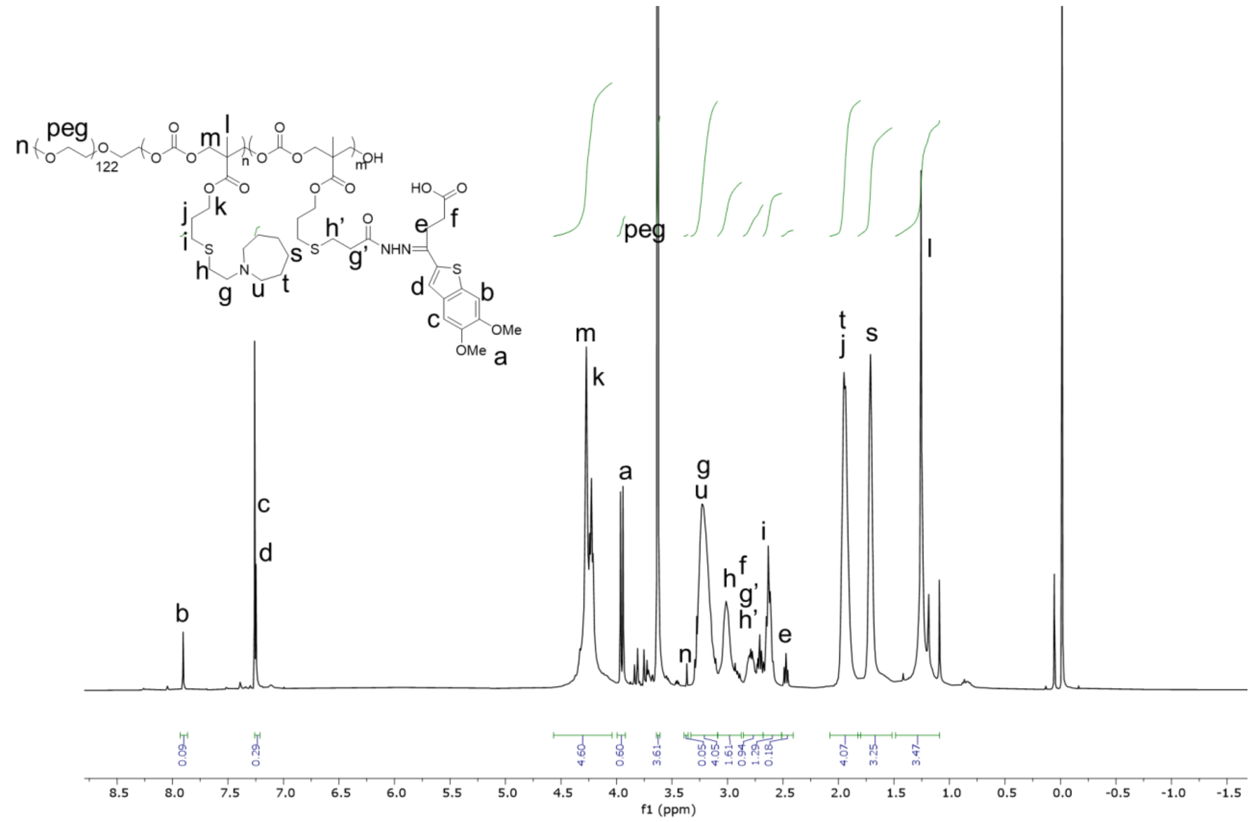

Supplementary Figure 4. ^{1}H NMR spectrum of PSC7A-pH-10MSA-2 copolymer.

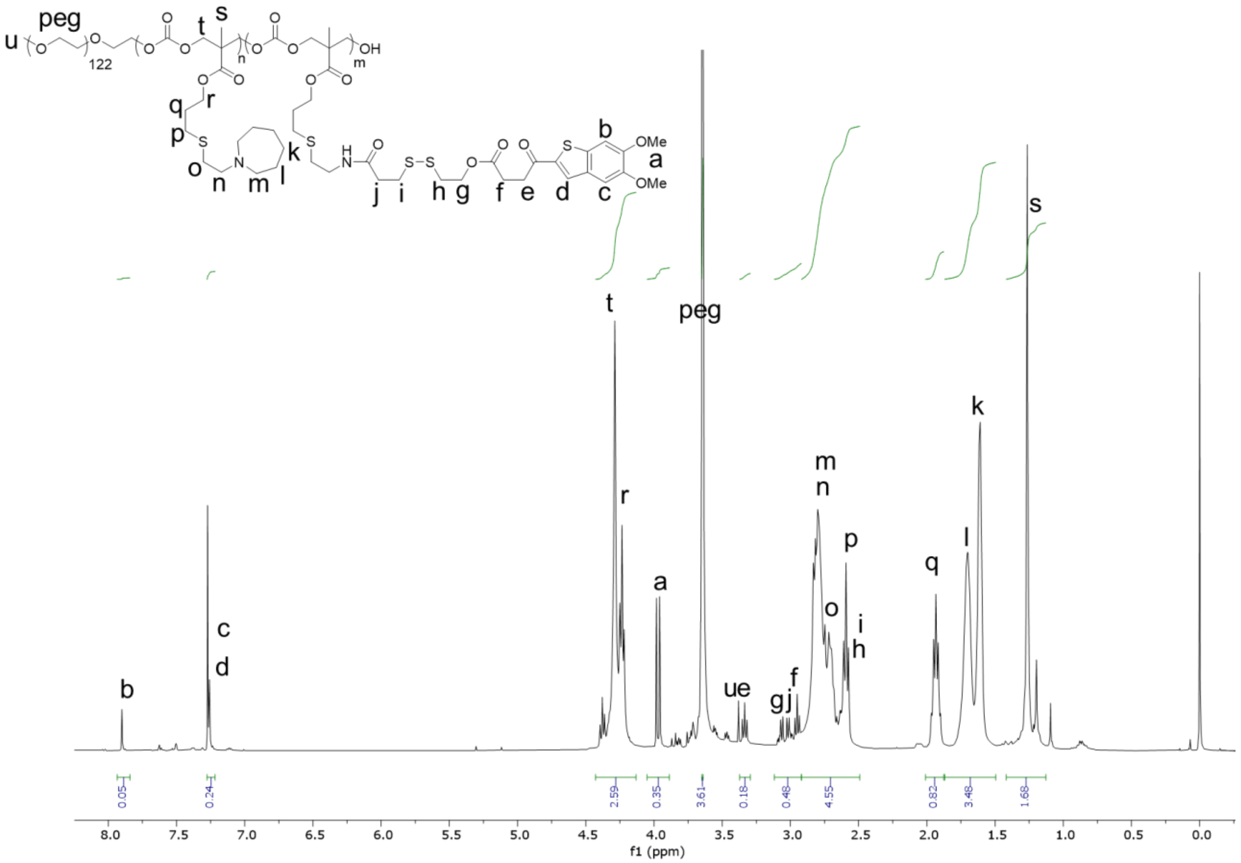

Supplementary Figure 5. ^{1}H NMR spectrum of PSC7A-Re-10MSA-2 copolymer.

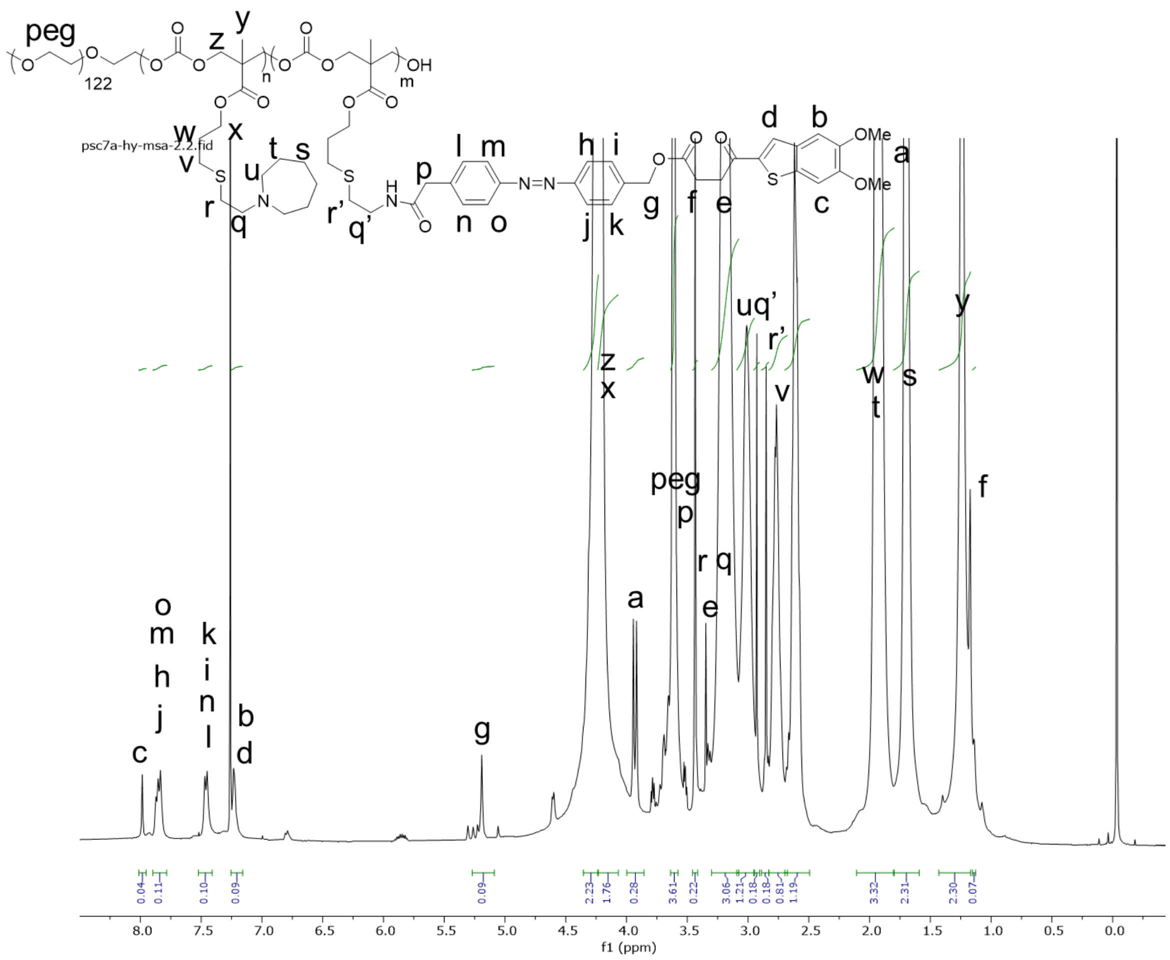

Supplementary Figure 6. ^1H NMR spectrum of PSC7A-Hy-10MSA-2 copolymer.

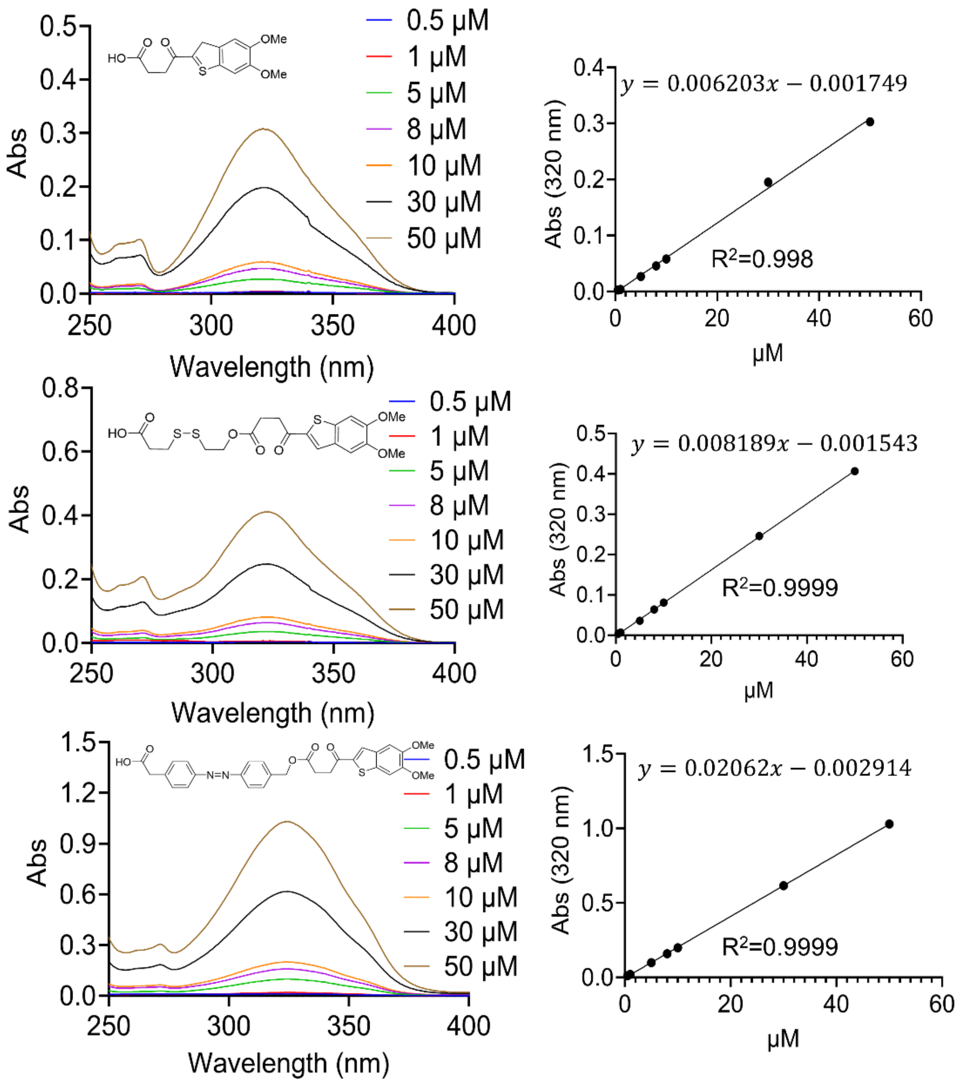

Supplementary Figure 7. UV-Vis spectra of precursor compounds and standard curves for MSA-2 quantification in polymers. UV-Vis absorption spectra of precursor compounds used in NP synthesis (left panel). Standard calibration curves (right panel) were generated to quantify the actual amount of MSA-2 conjugated to different polymer formulations.

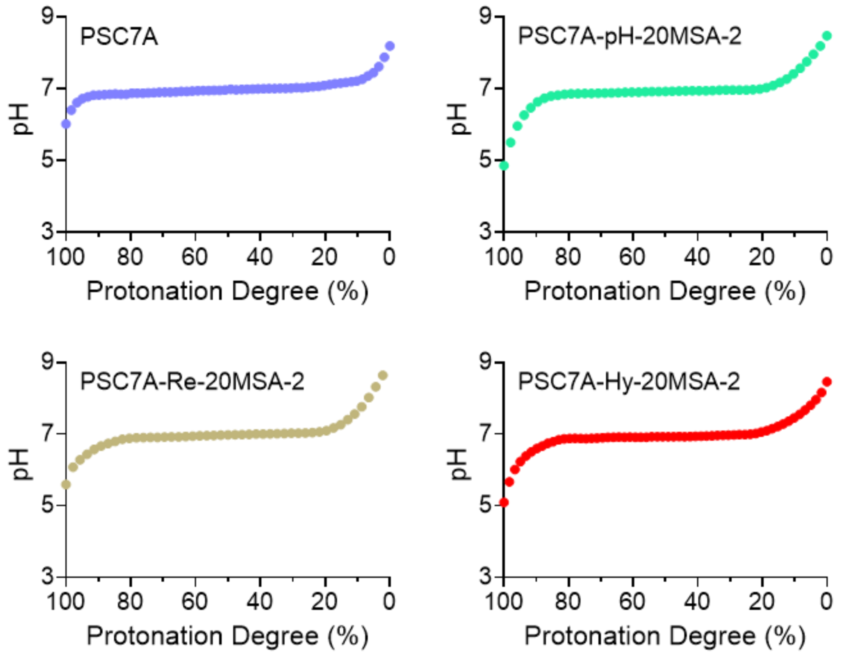

Supplementary Figure 8. pH titration cruves of PSC7A, PSC7A-pH-20MSA-2, PSC7A-Re-20MSA-2, and PSC7A-Hy-20MSA-2 copolymers. All the copolymers exhibit ultra-pH sensitivity and strong buffering capacity at their respective pKa values of 6.9.

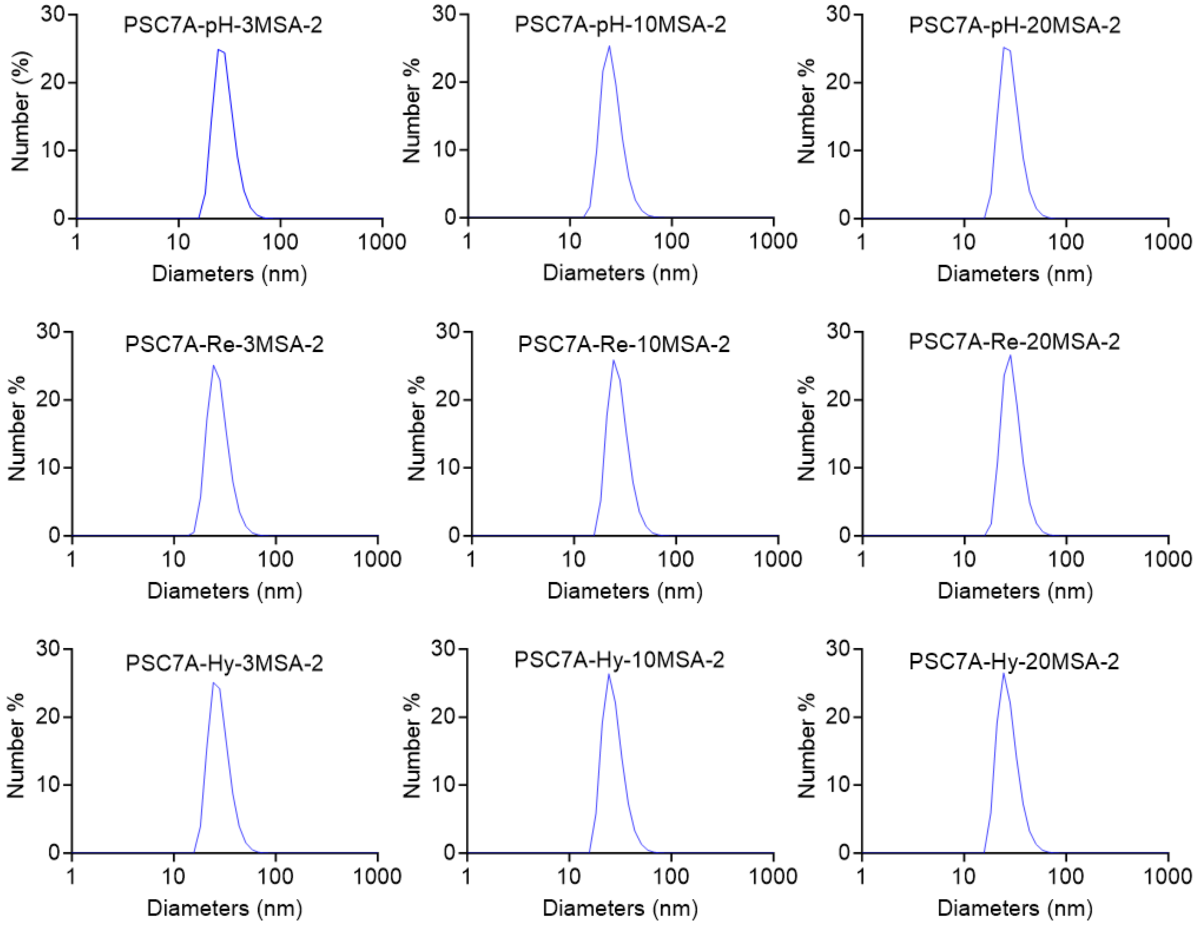

Supplementary Figure 9. Hydrodynamic size distribution of all NP formulations (1 mg/mL) in phosphate-buffered saline (pH 7.4) as measured by dynamic light scattering (DLS) analysis.

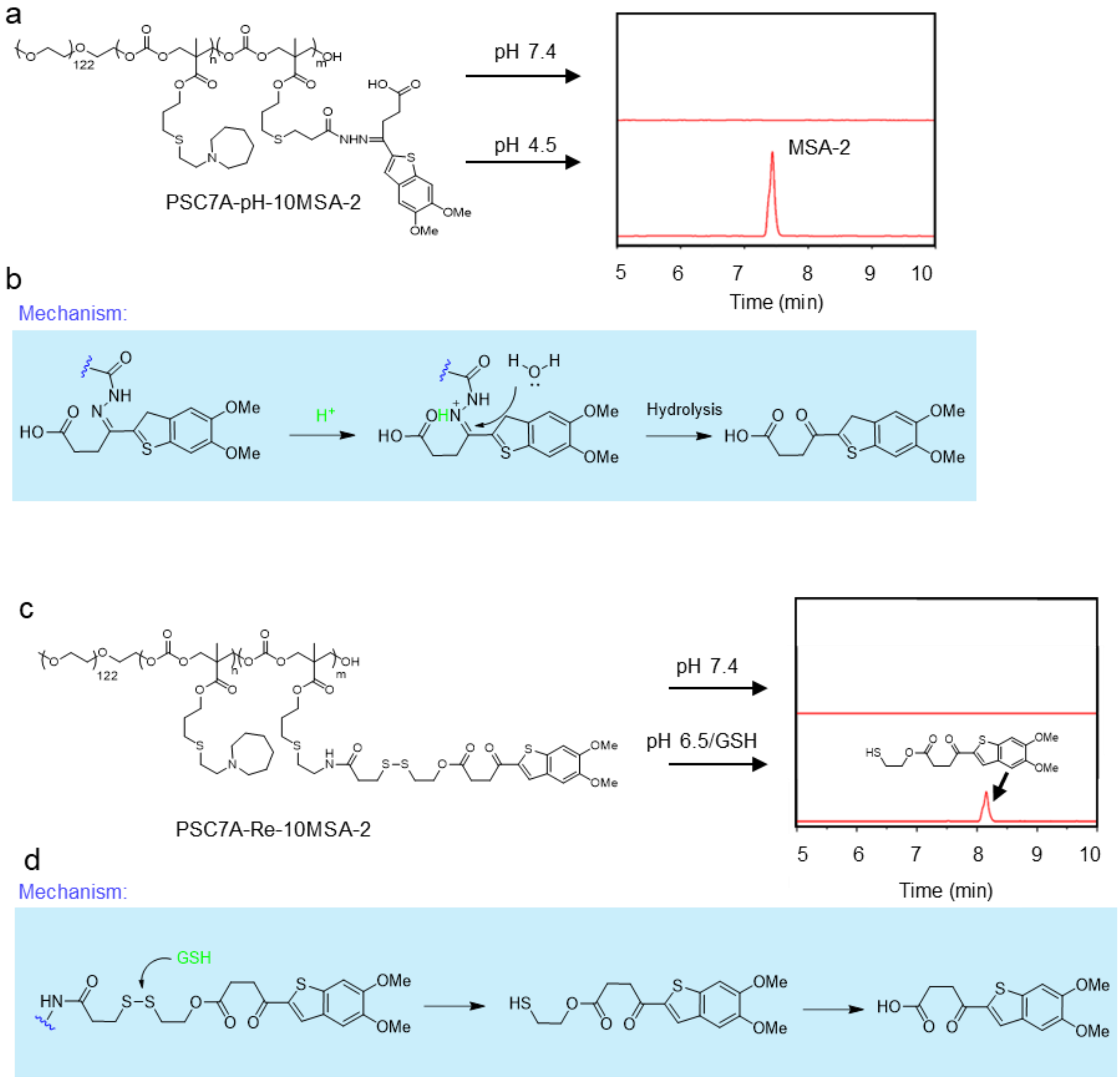

Supplementary Figure 10. a, HPLC spectrum of PSC7A-pH-10MSA-2 NPs after incubation in pH 4.5 PBS for 24 h at 37°C, showing pH-triggered drug release. **b**, Schematic of the acid-catalyzed hydrolysis of acyl hydrazone linker. **c**, HPLC spectrum of PSC7A-Re-10MSA-2 NPs after incubation in pH 6.5 PBS with 10 mM glutathione (GSH) for 24 h at 37°C, showing redox-responsive drug release. **d**, Schematic of the disulfide bond cleavage. In the cytoplasm (GSH ~10 mM), GSH induces thiol-disulfide exchange, leading to MSA-2 release.

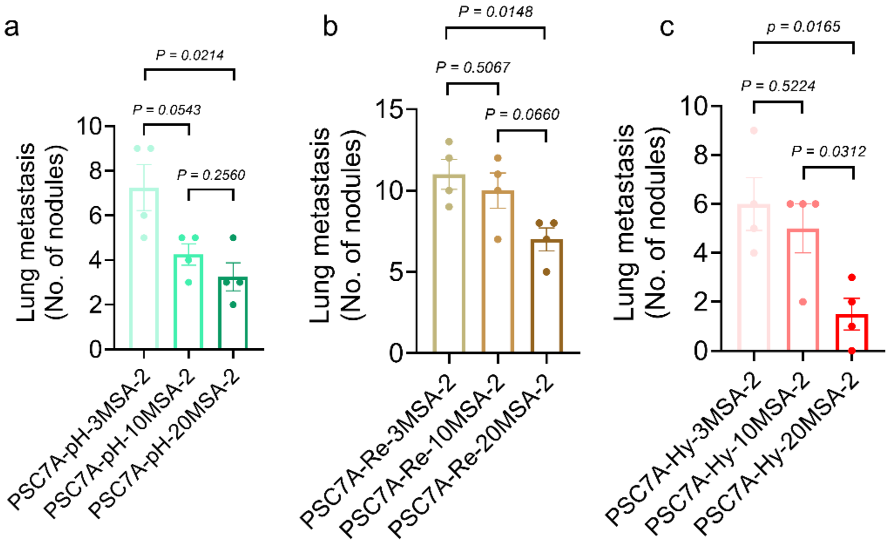

Supplementary Fig. 11. a-c, Statistical analysis among polymers with same linker but different DPRs. Each individual point represents the number of nodules in the lungs of mice after associated treatments. Statistical significance was analyzed by Student's two-tailed t test.

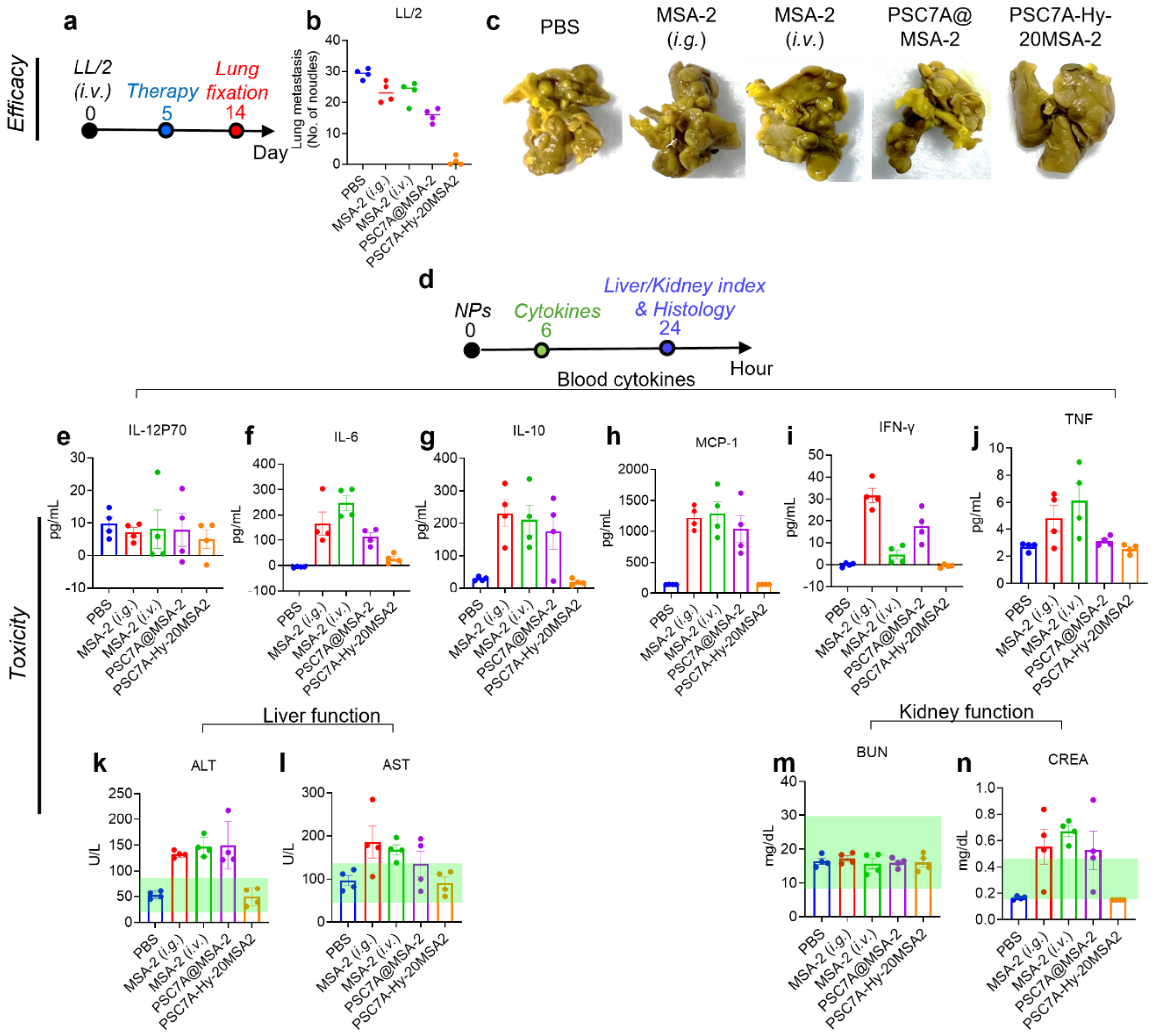

Supplementary Fig. 12: Antitumor efficacy and safety evaluation of different administration and formulation of STING-targeted therapy. a, Schematic illustration of the treatment design. **b–c**, Bar graph and representative lung images showing the number of metastatic nodules following treatment with PBS, MSA-2 (*i.g.*, 60 mg/kg), MSA-2 (*i.v.*, 2.3 mg/kg), PSC7A@MSA-2 (20 mg/kg), or PSC7A-Hy-20MSA-2 (PHM NP) (20 mg/kg). **d**, Experimental design for systemic toxicity assessment. **e–j**, Serum cytokine levels, including IL-12p70, IL-6, IL-10, IFN-γ, MCP-1, and TNF, measured after the indicated treatments (n = 4). **k–n**, Serum biochemistry indices (ALT, AST, BUN, and CREA) following administration of the same treatments; green shaded areas indicate the normal physiological range (n = 4).[1-4]

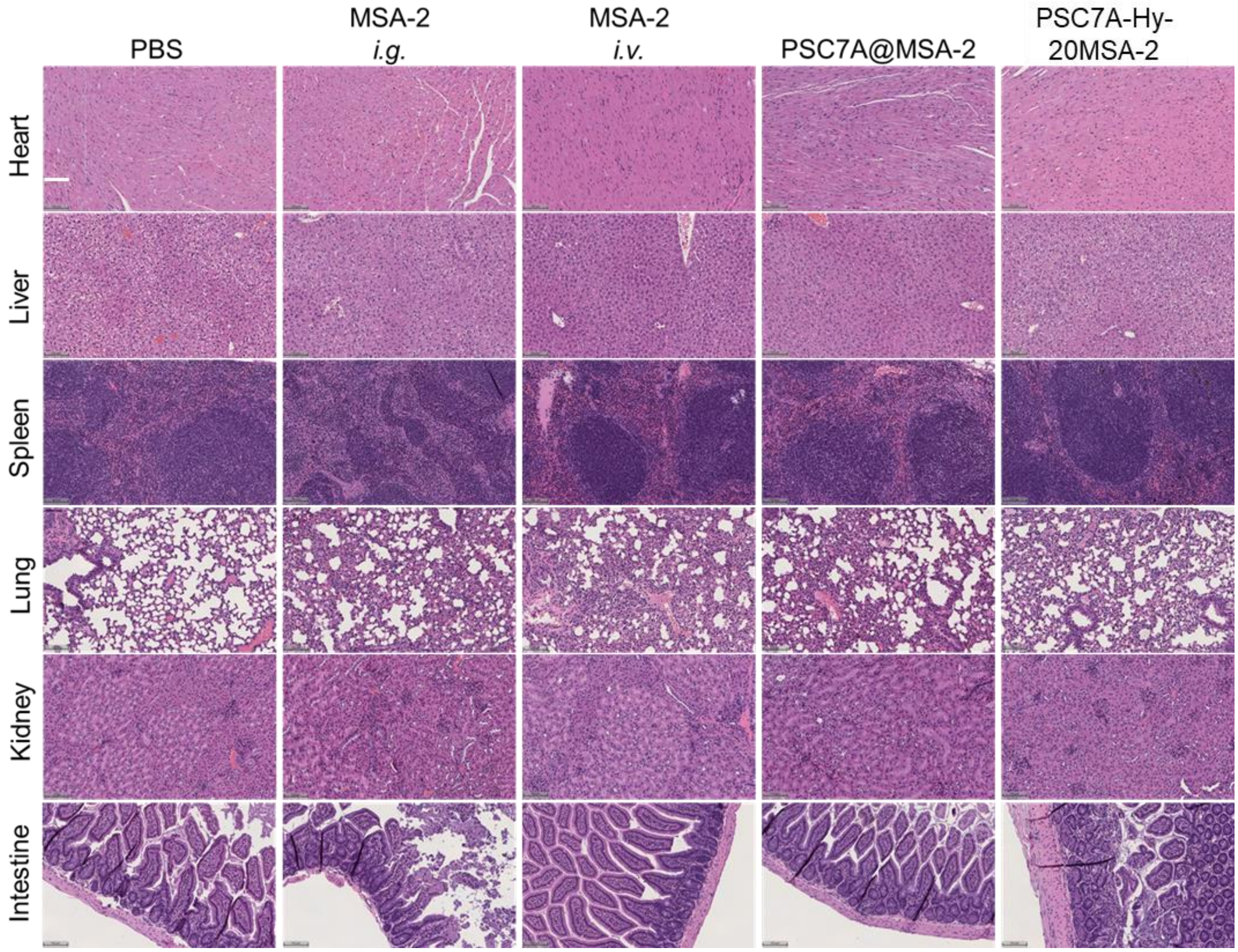

Supplementary Fig. 13: Histological evaluation of major organs after systemic treatments of different STING agonists. Representative H&E images of heart, liver, spleen, lung, kidney, and intestine collected 24 h after treatment with PBS, MSA-2 (*i.g.*, 60 mg/kg), MSA-2 (*i.v.*, 2.3 mg/kg), PSC7A@MSA-2 (20 mg/kg), or PHM NP (20 mg/kg). Scale bar, 100 μm.

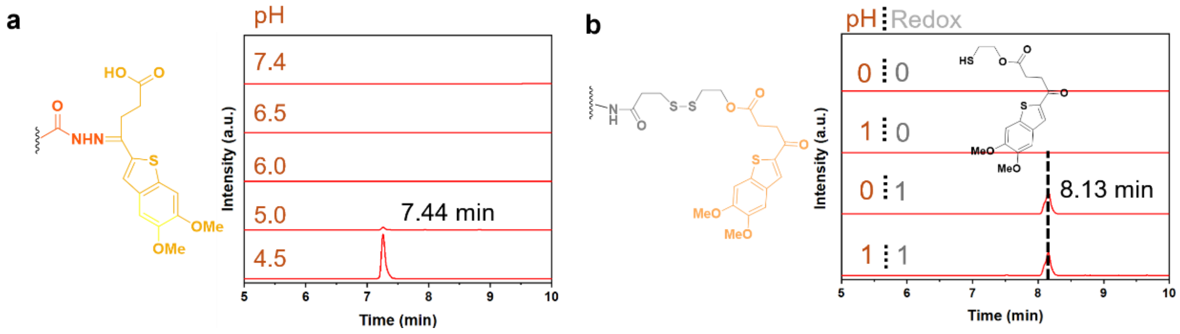

Supplementary Fig. 14. HPLC analysis of MSA-2 release from pH- and redox-sensitive polymer conjugates. a, Chromatograms of PSC7A–pH–MSA-2 (1 mg/mL) incubated in PBS at pH 7.4, 6.5, 6.0, 5.0, or 4.5 for 24 h at 37 °C. **b**, Chromatograms of PSC7A–Re–MSA-2 (1 mg/mL) incubated in PBS (pH 7.4 or 6.5 for 0 and 1 pH gate, specifically) with (1 in redox gate) or without (0 in redox gate) 10 mM GSH for 24 h at 37 °C.

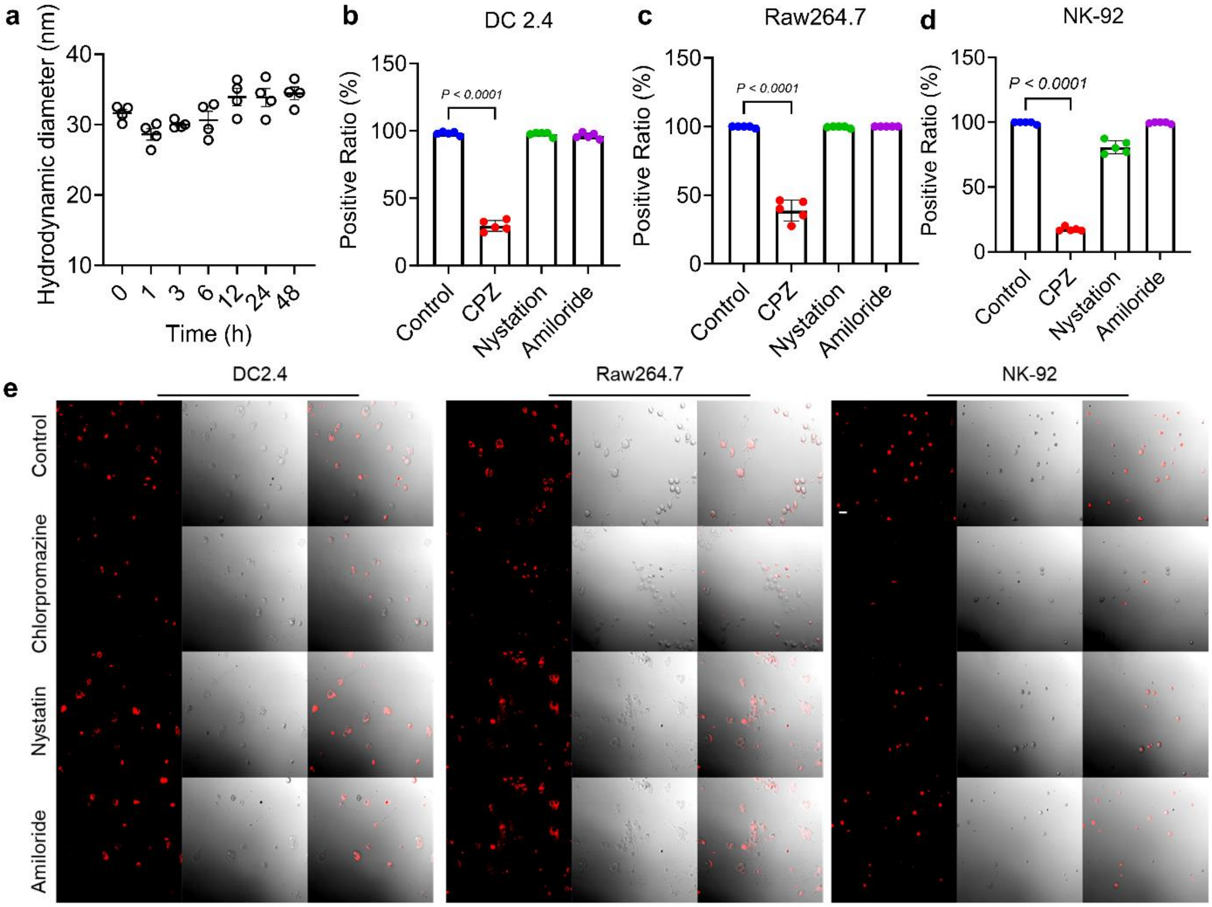

Supplementary Fig. 15: Micelle stability and cellular uptake mechanism of PHM NP. a, Hydrodynamic diameter of PHM NP (0.1/mg mL) was measured in cell culture DMEM medium at 37 °C over time (0, 1, 3, 6, 12, 24, and 48 h). **b–e**, For cell uptake mechanism of PHM NP, cells were pretreated with specific endocytosis inhibitors (chlorpromazine or CPZ, nystatin and amiloride) for 30 min prior to nanoparticle incubation. Cells were subsequently treated with PHM NP/PSC7A–Cy5 (0.1 mg/mL, mass ratio = 7:3), and nanoparticle uptake was quantified and compared among groups. Statistical significance was analyzed using one-way ANOVA. Scale bar, 20 μm.

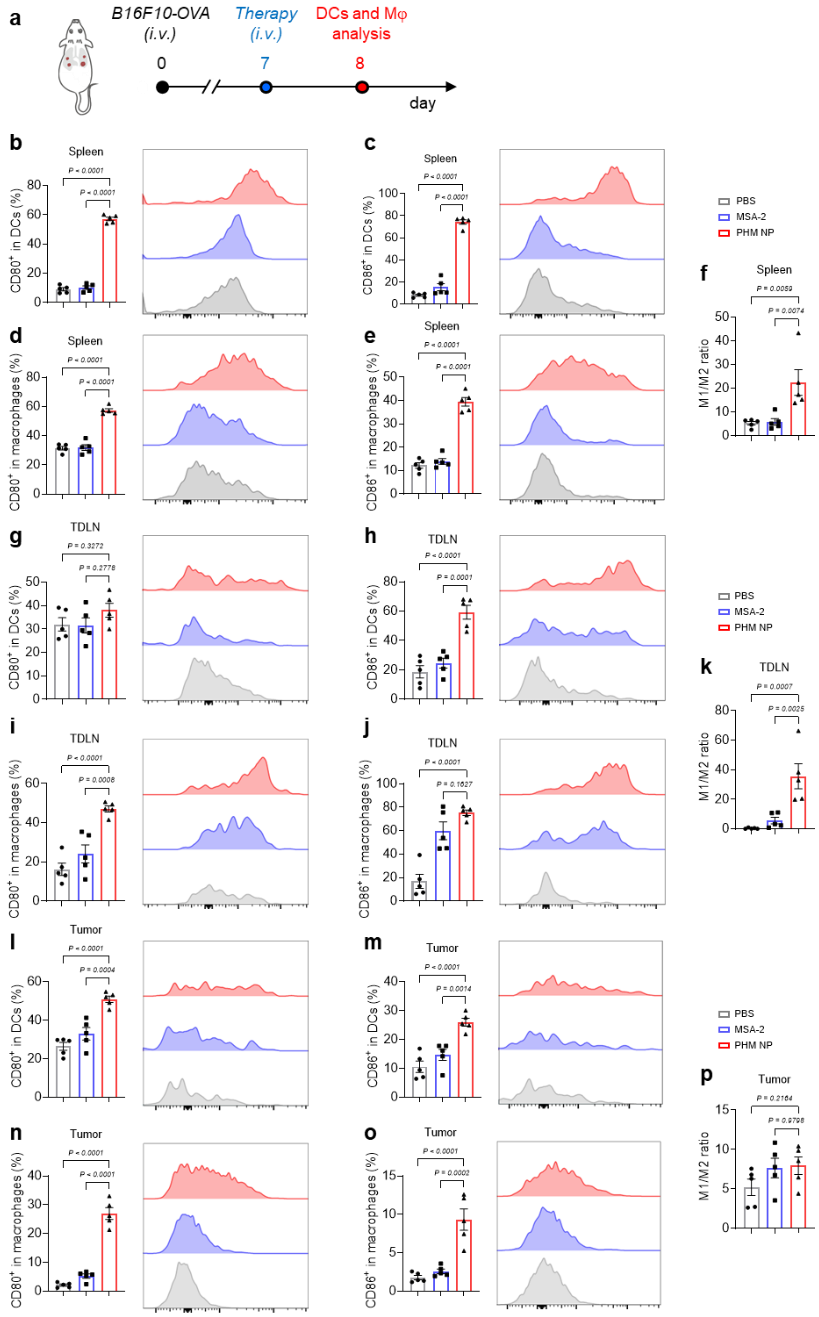

Supplementary Fig. 16. Activation of dendritic cells and macrophages following systemic STING activation. B16F10-OVA lung metastasis bearing mice were treated with PBS, free MSA-2 (2.3 mg/kg), or PHM NP (20 mg/kg, equivalent dose of MSA-2). After 24 h, spleen (**b-f**), TDLN (**g-k**), and tumor tissues (**l-p**) were harvested for flow-cytometric analysis of dendritic cell and macrophage activation markers (CD80, CD86) and M1/M2 polarization ratios. Statistical significance was determined by one-way ANOVA.

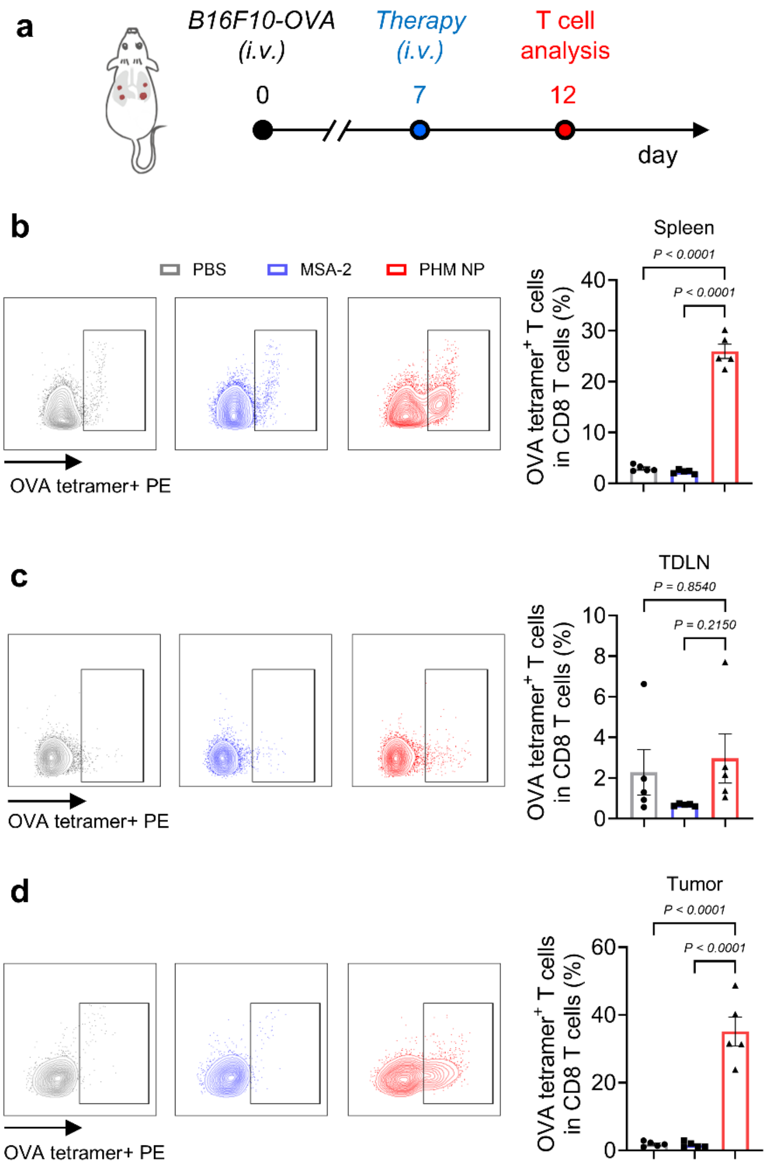

Supplementary Fig. 17. T-cell infiltration and antigen-specific CD8$^+$ T-cell response after systemic treatment. B16F10-OVA lung metastasis bearing mice were treated with PBS, free MSA-2 (2.3 mg/kg), or PHM NP (20 mg/kg.). Five days post-treatment, spleen (**b**), TDLN (**c**), and tumor tissues (**d**) were collected for flow-cytometric analysis of OVA-specific CD8$^+$ T cells using OVA-tetramer staining. Statistical significance was analyzed using one-way ANOVA.

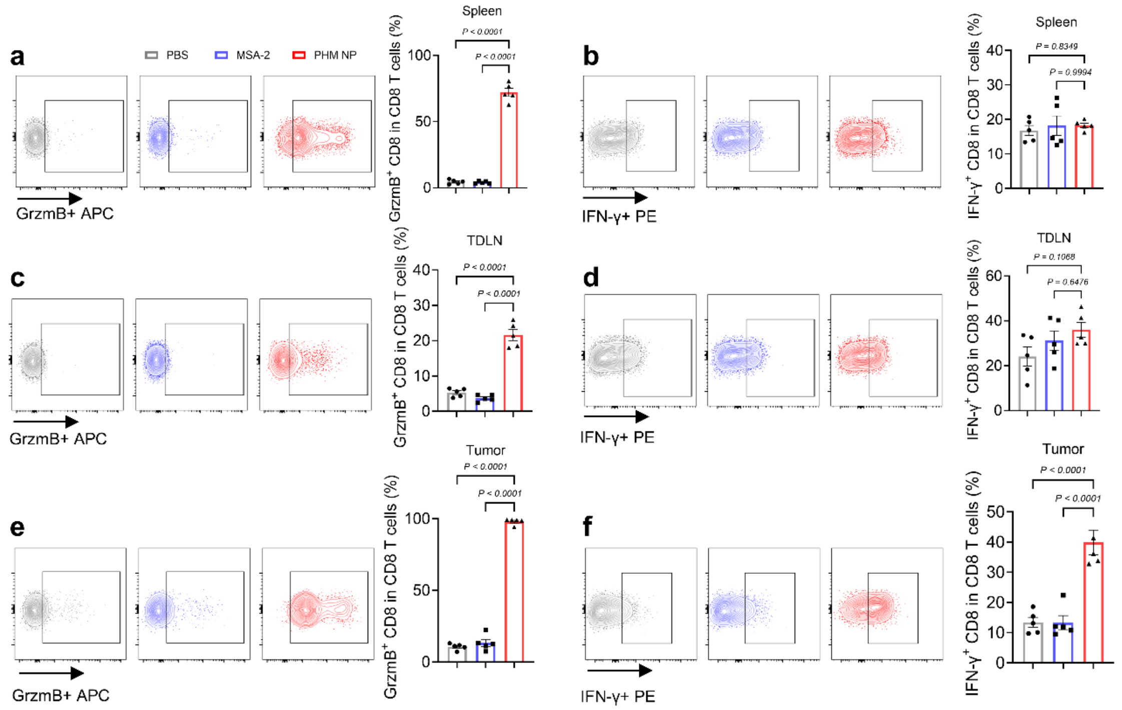

Supplementary Fig. 18. Activation of CD8+ T cells after systemic STING agonist treatment. B16F10-OVA lung metastasis bearing mice were treated with PBS, free MSA-2 (2.3 mg/kg), or PHM NP (20 mg/kg). At 18 h post-treatment, mice were intraperitoneally injected with 250 μg Brefeldin A to block cytokine secretion. At 24 h post-treatment, spleen (**a, b**), TDLN (**c, d**), and tumor tissues (**e, f**) were harvested for flow-cytometric analysis of intracellular granzyme B (GzmB) and IFN-γ expression in CD8+ T cells. Statistical significance was analyzed using one-way ANOVA.

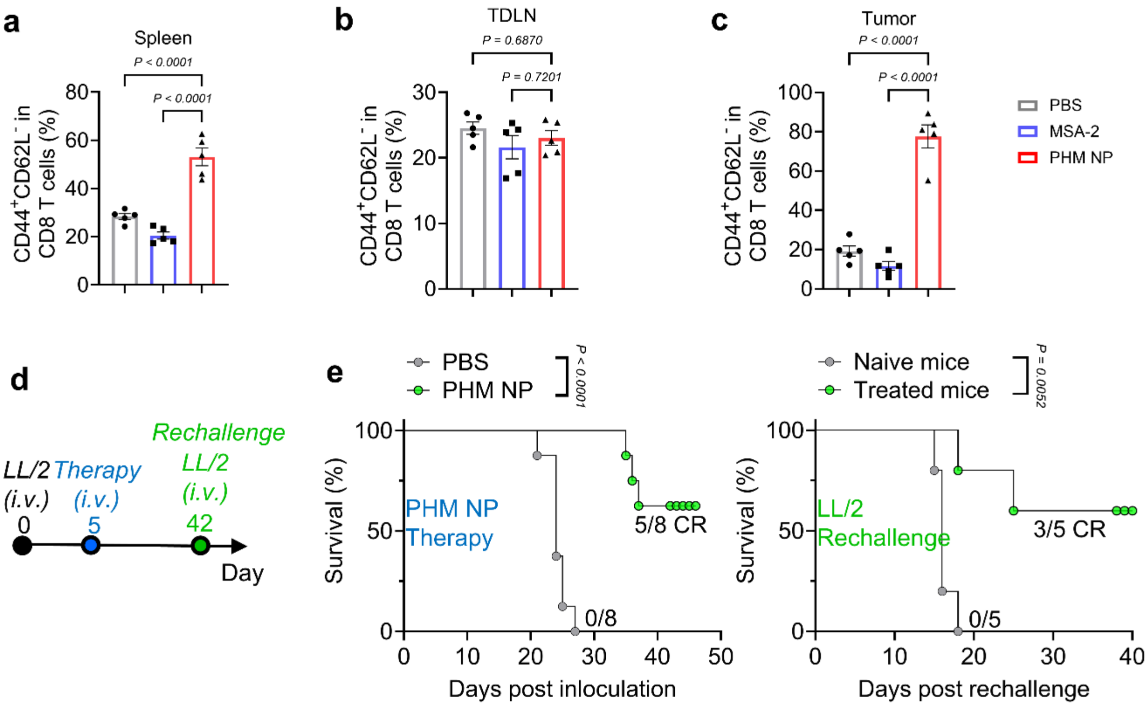

Supplementary Fig. 19. Memory T-cell formation and long-term antitumor immunity. a–c, B16F10-OVA lung metastasis–bearing mice were treated with PBS, free MSA-2 (2.3 mg/kg, *i.v.*), or PHM NP (20 mg/kg, *i.v.*). 5 days post-treatment, spleen, TDLN, and tumor tissues were collected for flow-cytometric analysis of effector/memory T-cell populations ($CD44^{+}CD62L^{-}$). **d**, Schematic illustration of the tumor rechallenge experiment. **e**, Tumor-free mice were subsequently rechallenged with LL/2 cells to evaluate long-term survival. Statistical significance was analyzed by one-way ANOVA (**a**–**c**) and the Mantel–Cox test (**e**).

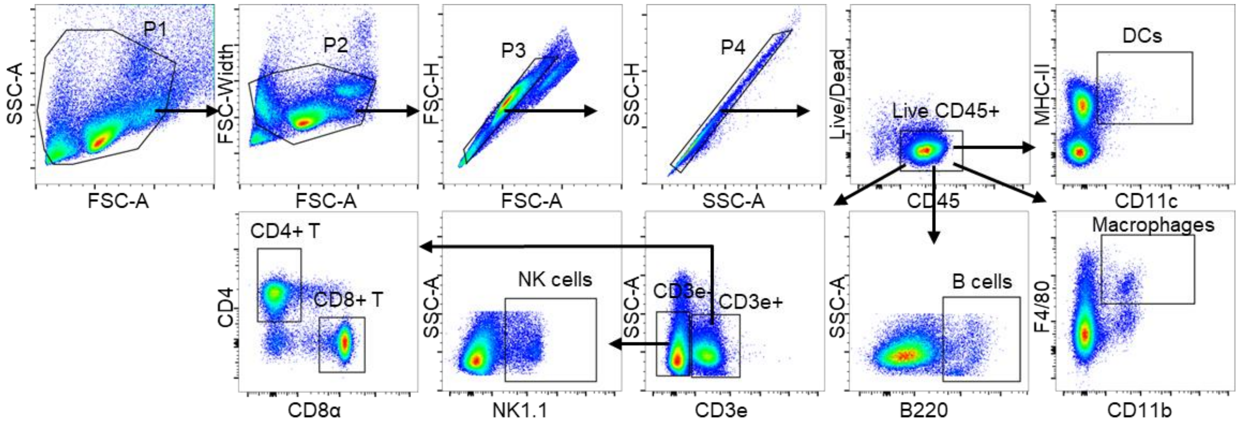

Supplementary Figure 20. Flow cytometry gating strategy for DC, macrophage, T, NK, and B cells analyses.

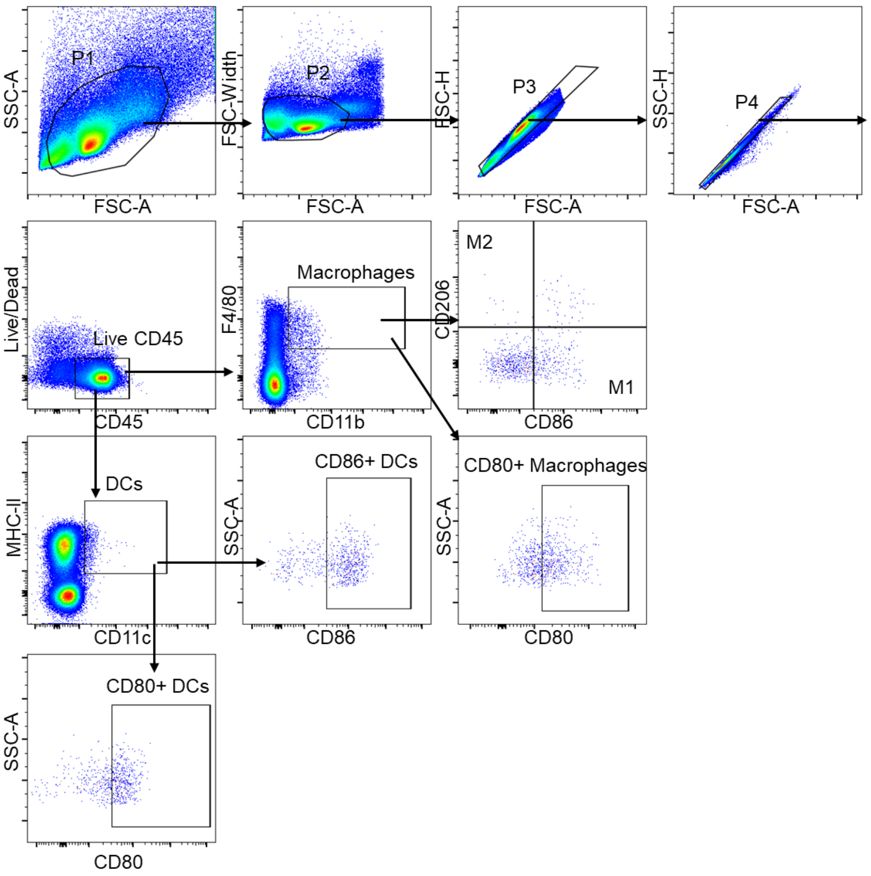

Supplementary Figure 21. Flow cytometry gating strategy for DC, macrophage activation analyses.

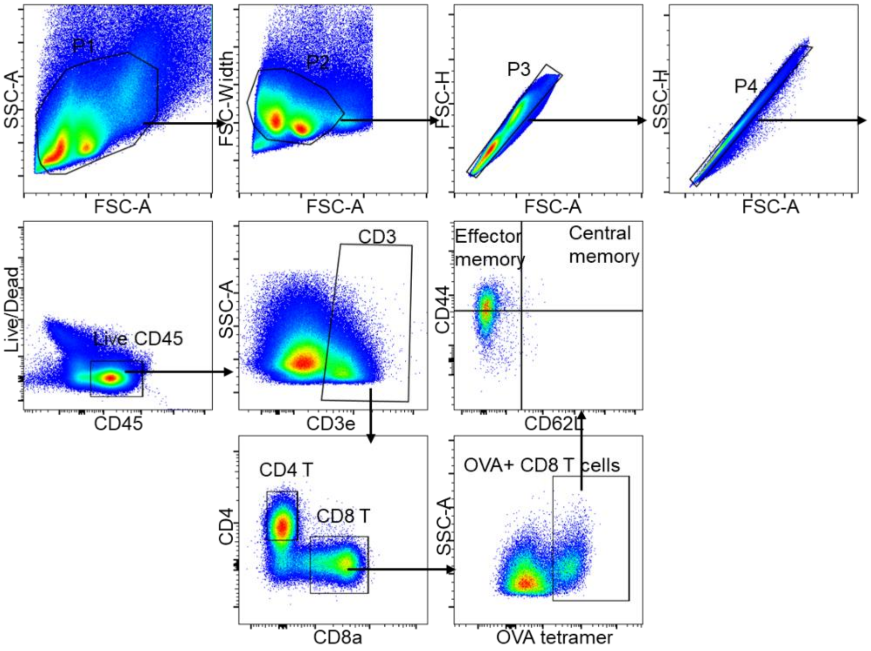

Supplementary Figure 22. Flow cytometry gating strategy for OVA-specific $CD8^+$ T cell analyses.

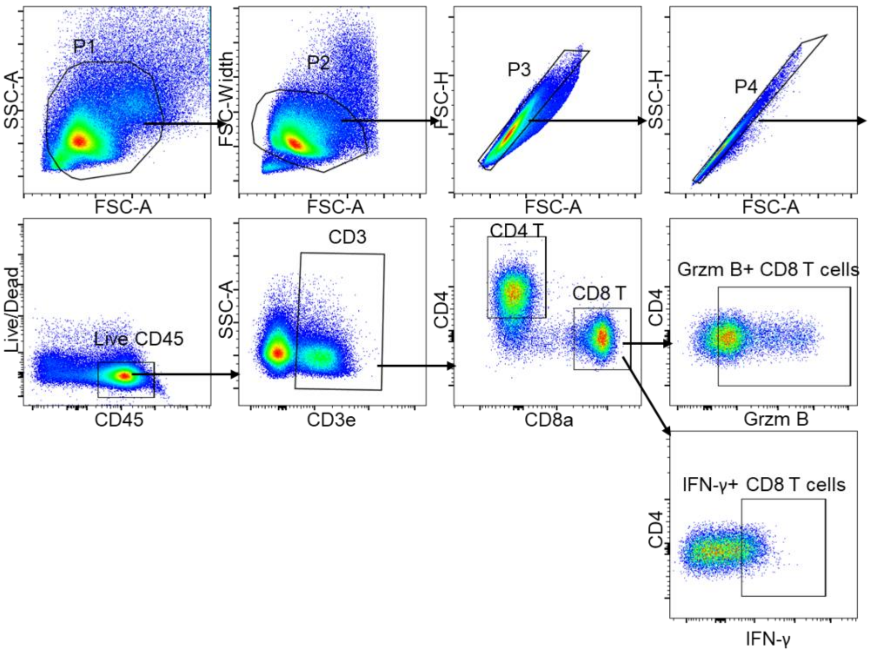

Supplementary Figure 23. Flow cytometry gating strategy for T cell activation analyses.

Supplementary Reference

1. Zhou, X. & Hansson, G. K. Effect of sex and age on serum biochemical reference ranges in C57BL/6J mice. *Comp. Med.* **54**, 176–178 (2004).
2. Jennette, J. C. *et al.* Anti–myeloperoxidase antibodies cause glomerulonephritis and vasculitis in mice. *J. Clin. Invest.* **110**, 955–963 (2002).
3. IDEXX BioAnalytics. *Mouse Clinical Pathology Reference Ranges.* IDEXX Laboratories, 2023.
4. Charles River Laboratories. *Mouse Hematology and Clinical Chemistry Reference Values (C57BL/6J).* Charles River Technical Bulletin, 2022.
