# Supplementary material for: AND logic nanoparticle for precision immunotherapy of metastatic cancers
Source: Nat Nanotechnol. 2026 Mar 9;21(4):606–16. doi: 10.1038/s41565-026-02130-3 (PMC13106039; doi:10.1038/s41565-026-02130-3)
Supplement: Supplementary file 2 — Reporting Summary [file 41565_2026_2130_MOESM2_ESM.pdf]

nature portfolio

Corresponding author(s): Jinming Gao

Last updated by author(s): Nov 1, 2025

Reporting Summary

Nature Portfolio wishes to improve the reproducibility of the work that we publish. This form provides structure for consistency and transparency in reporting. For further information on Nature Portfolio policies, see our Editorial Policies and the Editorial Policy Checklist.

Statistics

For all statistical analyses, confirm that the following items are present in the figure legend, table legend, main text, or Methods section.

n/a	Confirmed	
☐	☒	The exact sample size (*n*) for each experimental group/condition, given as a discrete number and unit of measurement
☐	☒	A statement on whether measurements were taken from distinct samples or whether the same sample was measured repeatedly
☐	☒	The statistical test(s) used AND whether they are one- or two-sided<br>*Only common tests should be described solely by name; describe more complex techniques in the Methods section.*
☒	☐	A description of all covariates tested
☐	☒	A description of any assumptions or corrections, such as tests of normality and adjustment for multiple comparisons
☐	☒	A full description of the statistical parameters including central tendency (e.g. means) or other basic estimates (e.g. regression coefficient) AND variation (e.g. standard deviation) or associated estimates of uncertainty (e.g. confidence intervals)
☐	☒	For null hypothesis testing, the test statistic (e.g. F, t, r) with confidence intervals, effect sizes, degrees of freedom and P value noted<br>*Give P values as exact values whenever suitable.*
☒	☐	For Bayesian analysis, information on the choice of priors and Markov chain Monte Carlo settings
☒	☐	For hierarchical and complex designs, identification of the appropriate level for tests and full reporting of outcomes
☒	☐	Estimates of effect sizes (e.g. Cohen's *d*, Pearson's *r*), indicating how they were calculated

Our web collection on statistics for biologists contains articles on many of the points above.

Software and code

Policy information about availability of computer code

Data collection	Bruker AN600 as used to collect HNMR data. Agilent 1260 HPLC machine coupled to a 6120 single quadrupole MS detectorwas used to collect LC-MS data. Malvern Zetasizer was used to collect the size data. SHIMADZU UV-1800 was used to collect UV-vis spectrum. METLER TOLEDO was used to collect the pH titration data. BioTek SYNERGY H1 microplate reader was used to collect to IFN-b data. BECKMAN COULTER was used to collect flowcytometry data. GelDoc Go Gel Imaging System (Bio-Rad) was used to collect western blot data. UTSW Metabolic Phenotyping Core for safety study. Agilent 1260 Infinity II was used to collect drug release data. Pearl Trilogy Small Animal Imaging System was used to collect PK and BD data. Akoya Biosciences Vectra Polaris™ was used to collect mIHC data. Hamamatsu Nanozoomer S60 was used to collect H&E data.
Data analysis	GraphPad Prism (v9.0.0) was used for plotting and statistical analysis. MestReNova was used for HNMR analysis. CytExpert (v2.4) and flowjo was used for analyzing flow cytometry data cytometry data. Halo image analysis software was used for mIHC analysis. NDP.view 2 was used for H&E analysis.

For manuscripts utilizing custom algorithms or software that are central to the research but not yet described in published literature, software must be made available to editors and reviewers. We strongly encourage code deposition in a community repository (e.g. GitHub). See the Nature Portfolio guidelines for submitting code & software for further information.

Data

Policy information about availability of data

All manuscripts must include a data availability statement. This statement should provide the following information, where applicable:

- Accession codes, unique identifiers, or web links for publicly available datasets
- A description of any restrictions on data availability
- For clinical datasets or third party data, please ensure that the statement adheres to our policy

All data supporting the findings of this study are included in the paper and its Supplementary Information. Source Data are provided with this paper.

Research involving human participants, their data, or biological material

Policy information about studies with human participants or human data. See also policy information about sex, gender (identity/presentation), and sexual orientation and race, ethnicity and racism.

Reporting on sex and gender	N.A.
Reporting on race, ethnicity, or other socially relevant groupings	N.A.
Population characteristics	N.A.
Recruitment	N.A.
Ethics oversight	N.A.

Note that full information on the approval of the study protocol must also be provided in the manuscript.

Field-specific reporting

Please select the one below that is the best fit for your research. If you are not sure, read the appropriate sections before making your selection.

[x] Life sciences [ ] Behavioural & social sciences [ ] Ecological, evolutionary & environmental sciences

For a reference copy of the document with all sections, see nature.com/documents/nr-reporting-summary-flat.pdf

Life sciences study design

All studies must disclose on these points even when the disclosure is negative.

Sample size	Given the current limitations in predicting the variation between animals, statistical methods were not used to pre-determine the sample sizes. Instead, sample sizes were selected to ensure the reproducibility of experiments while adhering to the principles of animal ethics regulation, specifically the concepts of replacement, reduction, and refinement. The sample sizes used in this study are consistent with those reported in similar previous studies. Each treatment group included at least three animals, which is the minimum required to achieve statistical significance ($p < 0.05$) between groups.
Data exclusions	No exclusion data.
Replication	The results of the experiments were consistent and reproducible, with each experiment performed a minimum of twice. Replication of the experiments is indicated in the figure captions.
Randomization	All samples and mice were randomly distributed into groups.
Blinding	This study did not employ blinding.

Reporting for specific materials, systems and methods

We require information from authors about some types of materials, experimental systems and methods used in many studies. Here, indicate whether each material, system or method listed is relevant to your study. If you are not sure if a list item applies to your research, read the appropriate section before selecting a response.

Materials & experimental systems

n/a	Involved in the study	
☐	☒	Antibodies
☐	☒	Eukaryotic cell lines
☐	☐	Palaeontology and archaeology
☐	☒	Animals and other organisms
☐	☐	Clinical data
☐	☐	Dual use research of concern
☐	☐	Plants

Methods

n/a	Involved in the study	
☐	☐	ChIP-seq
☐	☒	Flow cytometry
☐	☐	MRI-based neuroimaging

Antibodies

Antibodies used

Phospho-STING (72971, Cell signaling)
Purified anti-mouse/rat XCR1 Antibody (148202, Biolegend)
CD8α (98941, Cell signaling)
anti-NQO1 (N5288, Sigma, 1:800)
β-actin (A2228, Sigma/Aldrich, mouse monoclonal antibody, 1:5000)
CD45 PerCP (clone 30-F11, BioLegend)
MHC-II AF700 (clone M5/114.15.2, Invitrogen)
CD11c BV605 (clone N418, BioLegend)
CD11b BV605 (clone M1/70, BioLegend)
CD4 FITC (clone RM4-5, BioLegend)
CD8a PE (clone 53-6.7, BioLegend)
CD3e BV786 (clone 145-2C11, BD Biosciences)
F4/80 PE (clone BM8, Miltenyi Biotec)
NK1.1 PE-Cy7 (clone PK136, BD Biosciences)
B220 APC-Cy7 (clone RA3-6B2, BioLegend)
The LIVE/DEAD Fixable Aqua Dead Cell Stain Kit (Invitrogen, L34966)
anti-NK1.1 antibody (clone PK136, BioXcell)
anti-CD8a antibody (clone YTS169.4, BioXcell)
anti-CD4 antibody (clone GK1.5, BioXcell)
CD11b PB (clone M1/70, BioLegend)
F4/80 APC-Cy7 (clone BM8, BioLegend)
CD80 PE-Cy7 (clone 16-10A1, BioLegend)
CD86 APC (clone GL-1, BioLegend)
CD206 PE (clone)
CD4 PB (clone GK1.5, BioLegend)
CD8a AF700 (clone QA17A07, BioLegend)
CD3e BV786 (clone 145-2C11, BD Biosciences)
H-2Kb OVA tetramer PE (SIINFEKL, MBL Life Sciences)
CD62L PE-Cy7 (clone MEL-14, BioLegend)
and CD44 APC-Cy7 (clone IM7, BioLegend)
Grzm B APC (clone QA16A02, BioLegend)
IFN-γ PE (clone XMG1.2, BioLegend)
The LIVE/DEAD Fixable Aqua Dead Cell Stain Kit (Invitrogen, L34966)

Validation

Phospho-STING (72971, Cell signaling) for IHC. Reactivity with mouse was tested.
Purified anti-mouse/rat XCR1 Antibody (148202, Biolegend) for IHC. Reactivity with mouse was tested.
CD8α (98941, Cell signaling) for IHC. Reactivity with mouse was tested.
anti-NQO1 (N5288, Sigma, 1:800) for WB. Reactivity with mouse was tested.
β-actin (A2228, Sigma/Aldrich, mouse monoclonal antibody, 1:5000) for WB. Reactivity with mouse was tested.
CD45 PerCP (clone 30-F11, BioLegend) for flow cytometry. Reactivity with mouse was tested.
MHC-II AF700 (clone M5/114.15.2, Invitrogen) for flow cytometry. Reactivity with mouse was tested.
CD11c BV605 (clone N418, BioLegend) for flow cytometry. Reactivity with mouse was tested.
CD11b BV605 (clone M1/70, BioLegend) for flow cytometry. Reactivity with mouse was tested.
CD4 FITC (clone RM4-5, BioLegend) for flow cytometry. Reactivity with mouse was tested.
CD8a PE (clone 53-6.7, BioLegend) for flow cytometry. Reactivity with mouse was tested.
CD3e BV786 (clone 145-2C11, BD Biosciences) for flow cytometry. Reactivity with mouse was tested.
F4/80 PE (clone BM8, Miltenyi Biotec) for flow cytometry. Reactivity with mouse was tested.
NK1.1 PE-Cy7 (clone PK136, BD Biosciences) for flow cytometry. Reactivity with mouse was tested.
B220 APC-Cy7 (clone RA3-6B2, BioLegend) for flow cytometry. Reactivity with mouse was tested.
anti-NK1.1 antibody (clone PK136, BioXcell) for in vivo cell depletion. Reactivity with mouse was tested.
anti-CD8a antibody (clone YTS169.4, BioXcell) for in vivo cell depletion. Reactivity with mouse was tested.
anti-CD4 antibody (clone GK1.5, BioXcell) for in vivo cell depletion. Reactivity with mouse was tested.
CD11b PB (clone M1/70, BioLegend) for flow cytometry. Reactivity with mouse was tested.
F4/80 APC-Cy7 (clone BM8, BioLegend) for flow cytometry. Reactivity with mouse was tested.
CD80 PE-Cy7 (clone 16-10A1, BioLegend) for flow cytometry. Reactivity with mouse was tested.
CD86 APC (clone GL-1, BioLegend) for flow cytometry. Reactivity with mouse was tested.
CD206 PE (clone) for flow cytometry. Reactivity with mouse was tested.
CD4 PB (clone GK1.5, BioLegend) for flow cytometry. Reactivity with mouse was tested.

CD8a AF700 (clone QA17A07, BioLegend) for flow cytometry. Reactivity with mouse was tested.
CD3e BV786 (clone 145-2C11, BD Biosciences) for flow cytometry. Reactivity with mouse was tested.
H-2Kb OVA tetramer PE (SIINFEKL, MBL Life Sciences) for flow cytometry. Reactivity with mouse was tested.
CD62L PE-Cy7 (clone MEL-14, BioLegend) for flow cytometry. Reactivity with mouse was tested.
and CD44 APC-Cy7 (clone IM7, BioLegend) for flow cytometry. Reactivity with mouse was tested.
Grzm B APC (clone QA16A02, BioLegend) for flow cytometry. Reactivity with mouse was tested.
IFN-γ PE (clone XMG1.2, BioLegend) for flow cytometry. Reactivity with mouse was tested.
The LIVE/DEAD Fixable Aqua Dead Cell Stain Kit (Invitrogen, L34966) for flow cytometry. Reactivity with mouse was tested.

Eukaryotic cell lines

Policy information about cell lines and Sex and Gender in Research

Cell line source(s)

THP1-Lucia ISG cells were purchased from InvivoGen (Catlog: thpl-isg). B16F10 cells were purchased from ATCC (Catlog: CRL-6475). 4T1 cells were provided by S. Huang, Massey Cancer Center, Virginia Commonwealth University. LL/2 were provided by Z. "James" Chen, University of Texas (UT) Southwestern Medical Center. (UT Southwestern). DC 2.4 were provided by Daniel J. Siegwart, UT Southwestern. Raw264.7 were provided by Z. "James" Chen. IMCD3 were provided by Yu Xun, UT Southwestern.

Authentication

The identity of the cell lines was routinely verified based on their morphological characteristics and initially authenticated through short tandem repeat (STR) profiling.

Mycoplasma contamination

Cells were routinely tested and confirmed to be free of mycoplasma contamination.

Commonly misidentified lines
(See ICLAC register)

This study did not use any commonly misidentified cell lines.

Palaeontology and Archaeology

Specimen provenance

Provide provenance information for specimens and describe permits that were obtained for the work (including the name of the issuing authority, the date of issue, and any identifying information). Permits should encompass collection and, where applicable, export.

Specimen deposition

Indicate where the specimens have been deposited to permit free access by other researchers.

Dating methods

If new dates are provided, describe how they were obtained (e.g. collection, storage, sample pretreatment and measurement), where they were obtained (i.e. lab name), the calibration program and the protocol for quality assurance OR state that no new dates are provided.

☐ Tick this box to confirm that the raw and calibrated dates are available in the paper or in Supplementary Information.

Ethics oversight

Identify the organization(s) that approved or provided guidance on the study protocol, OR state that no ethical approval or guidance was required and explain why not.

Note that full information on the approval of the study protocol must also be provided in the manuscript.

Animals and other research organisms

Policy information about studies involving animals; ARRIVE guidelines recommended for reporting animal research, and Sex and Gender in Research

Laboratory animals

STING−/− and Batf3−/− mice were obtained from the Jackson Laboratory, while C57BL/6 wild-type (WT) and BALB/c mice were sourced from Charles River Laboratories. All mice were kept under specific pathogen-free conditions in a barrier facility with a 12-hour light–12-hour dark cycle, and fed standard chow (2916, Teklad Global). The housing room temperature ranged from 68°F to 79°F, with an average of 72°F, and humidity levels were maintained between 30% and 50%, averaging around 50%. The experimental groups consisted of randomly selected female littermates, approximately 6-8 weeks old, of the same strain.

Wild animals

Wild animals were not involved in this study

Reporting on sex

All mice used in this study were female.

Field-collected samples

Samples collected from the field were not used in this study.

Ethics oversight

All procedures were conducted in accordance with the ethical guidelines and protocols approved by the AAALAC-accredited Institutional Animal Care and Use Committee at UT Southwestern Medical Center under protocol number 2017-102331.

Note that full information on the approval of the study protocol must also be provided in the manuscript.

Clinical data

Policy information about clinical studies

All manuscripts should comply with the ICMJE guidelines for publication of clinical research and a completed CONSORT checklist must be included with all submissions.

Clinical trial registration	*Provide the trial registration number from ClinicalTrials.gov or an equivalent agency.*
Study protocol	*Note where the full trial protocol can be accessed OR if not available, explain why.*
Data collection	*Describe the settings and locales of data collection, noting the time periods of recruitment and data collection.*
Outcomes	*Describe how you pre-defined primary and secondary outcome measures and how you assessed these measures.*

Dual use research of concern

Policy information about dual use research of concern

Hazards

Could the accidental, deliberate or reckless misuse of agents or technologies generated in the work, or the application of information presented in the manuscript, pose a threat to:

No	Yes	
☐	☐	Public health
☐	☐	National security
☐	☐	Crops and/or livestock
☐	☐	Ecosystems
☐	☐	Any other significant area

Experiments of concern

Does the work involve any of these experiments of concern:

No	Yes	
☐	☐	Demonstrate how to render a vaccine ineffective
☐	☐	Confer resistance to therapeutically useful antibiotics or antiviral agents
☐	☐	Enhance the virulence of a pathogen or render a nonpathogen virulent
☐	☐	Increase transmissibility of a pathogen
☐	☐	Alter the host range of a pathogen
☐	☐	Enable evasion of diagnostic/detection modalities
☐	☐	Enable the weaponization of a biological agent or toxin
☐	☐	Any other potentially harmful combination of experiments and agents

Plants

Seed stocks	*Report on the source of all seed stocks or other plant material used. If applicable, state the seed stock centre and catalogue number. If plant specimens were collected from the field, describe the collection location, date and sampling procedures.*
Novel plant genotypes	*Describe the methods by which all novel plant genotypes were produced. This includes those generated by transgenic approaches, gene editing, chemical/radiation-based mutagenesis and hybridization. For transgenic lines, describe the transformation method, the number of independent lines analyzed and the generation upon which experiments were performed. For gene-edited lines, describe the editor used, the endogenous sequence targeted for editing, the targeting guide RNA sequence (if applicable) and how the editor was applied.*
Authentication	*Describe any authentication procedures for each seed stock used or novel genotype generated. Describe any experiments used to assess the effect of a mutation and, where applicable, how potential secondary effects (e.g. second site T-DNA insertions, mosiacism, off-target gene editing) were examined.*

ChIP-seq

Data deposition

☐ Confirm that both raw and final processed data have been deposited in a public database such as GEO.

☐ Confirm that you have deposited or provided access to graph files (e.g. BED files) for the called peaks.

Data access links<br>*May remain private before publication.*	*For "Initial submission" or "Revised version" documents, provide reviewer access links. For your "Final submission" document, provide a link to the deposited data.*
Files in database submission	*Provide a list of all files available in the database submission.*
Genome browser session<br>(e.g. UCSC)	*Provide a link to an anonymized genome browser session for "Initial submission" and "Revised version" documents only, to enable peer review. Write "no longer applicable" for "Final submission" documents.*

Methodology

Replicates	*Describe the experimental replicates, specifying number, type and replicate agreement.*
Sequencing depth	*Describe the sequencing depth for each experiment, providing the total number of reads, uniquely mapped reads, length of reads and whether they were paired- or single-end.*
Antibodies	*Describe the antibodies used for the ChIP-seq experiments; as applicable, provide supplier name, catalog number, clone name, and lot number.*
Peak calling parameters	*Specify the command line program and parameters used for read mapping and peak calling, including the ChIP, control and index files used.*
Data quality	*Describe the methods used to ensure data quality in full detail, including how many peaks are at FDR 5% and above 5-fold enrichment.*
Software	*Describe the software used to collect and analyze the ChIP-seq data. For custom code that has been deposited into a community repository, provide accession details.*

Flow Cytometry

Plots

Confirm that:

☒ The axis labels state the marker and fluorochrome used (e.g. CD4-FITC).

☒ The axis scales are clearly visible. Include numbers along axes only for bottom left plot of group (a 'group' is an analysis of identical markers).

☒ All plots are contour plots with outliers or pseudocolor plots.

☒ A numerical value for number of cells or percentage (with statistics) is provided.

Methodology

Sample preparation	Mice were euthanized 24 hours post-injection, and tissues, including lung metastases/tumors, tdLNs, and spleens, were collected and processed into single-cell suspensions. Cells were stained with fluorochrome-conjugated antibodies.
Instrument	Beckman CytoFLEX flow cytometers
Software	CytExpert v2.4, flowJo
Cell population abundance	500000 events were collected in each analysis.
Gating strategy	Briefly, cells were gated on FSC-A/SSC-A.

☐ Tick this box to confirm that a figure exemplifying the gating strategy is provided in the Supplementary Information.

Magnetic resonance imaging

Experimental design

Design type	*Indicate task or resting state; event-related or block design.*
Design specifications	*Specify the number of blocks, trials or experimental units per session and/or subject, and specify the length of each trial*

Design specifications	*or block (if trials are blocked) and interval between trials.*
Behavioral performance measures	*State number and/or type of variables recorded (e.g. correct button press, response time) and what statistics were used to establish that the subjects were performing the task as expected (e.g. mean, range, and/or standard deviation across subjects).*

Acquisition

Imaging type(s)	*Specify: functional, structural, diffusion, perfusion.*
Field strength	*Specify in Tesla*
Sequence & imaging parameters	*Specify the pulse sequence type (gradient echo, spin echo, etc.), imaging type (EPI, spiral, etc.), field of view, matrix size, slice thickness, orientation and TE/TR/flip angle.*
Area of acquisition	*State whether a whole brain scan was used OR define the area of acquisition, describing how the region was determined.*

Diffusion MRI ☐ Used ☒ Not used

Preprocessing

Preprocessing software	*Provide detail on software version and revision number and on specific parameters (model/functions, brain extraction, segmentation, smoothing kernel size, etc.).*
Normalization	*If data were normalized/standardized, describe the approach(es): specify linear or non-linear and define image types used for transformation OR indicate that data were not normalized and explain rationale for lack of normalization.*
Normalization template	*Describe the template used for normalization/transformation, specifying subject space or group standardized space (e.g. original Talairach, MNI305, ICBM152) OR indicate that the data were not normalized.*
Noise and artifact removal	*Describe your procedure(s) for artifact and structured noise removal, specifying motion parameters, tissue signals and physiological signals (heart rate, respiration).*
Volume censoring	*Define your software and/or method and criteria for volume censoring, and state the extent of such censoring.*

Statistical modeling & inference

Model type and settings	*Specify type (mass univariate, multivariate, RSA, predictive, etc.) and describe essential details of the model at the first and second levels (e.g. fixed, random or mixed effects; drift or auto-correlation).*
Effect(s) tested	*Define precise effect in terms of the task or stimulus conditions instead of psychological concepts and indicate whether ANOVA or factorial designs were used.*

Specify type of analysis: ☐ Whole brain ☐ ROI-based ☐ Both

Statistic type for inference<br>(See Eklund et al. 2016)	*Specify voxel-wise or cluster-wise and report all relevant parameters for cluster-wise methods.*
Correction	*Describe the type of correction and how it is obtained for multiple comparisons (e.g. FWE, FDR, permutation or Monte Carlo).*

Models & analysis

n/a	Involved in the study
☐	☐ Functional and/or effective connectivity
☐	☐ Graph analysis
☐	☐ Multivariate modeling or predictive analysis

Functional and/or effective connectivity	*Report the measures of dependence used and the model details (e.g. Pearson correlation, partial correlation, mutual information).*
Graph analysis	*Report the dependent variable and connectivity measure, specifying weighted graph or binarized graph, subject- or group-level, and the global and/or node summaries used (e.g. clustering coefficient, efficiency, etc.).*
Multivariate modeling and predictive analysis	*Specify independent variables, features extraction and dimension reduction, model, training and evaluation metrics.*
